# Protection and Analysis of Intangible Cultural Heritage Videos Based on Keyframe Extraction and Adaptive Weight Assignment

**DOI:** 10.1371/journal.pone.0330176

**Published:** 2025-08-29

**Authors:** Qingbin Hou

**Affiliations:** School of Fashion Art, Shaanxi University of International Trade & Commerce, Xi’an, China; Dayananda Sagar University, INDIA

## Abstract

To preserve the intangible cultural heritage digitally and effectively manage and analyze the intangible cultural heritage video data, the research creatively employs target recognition algorithms and keyframe extraction to perform video extraction and analysis. The keyframe extraction and target detection model is constructed with the help of shot boundary detection, feature pyramid network, and attention mechanism. The experimental results revealed that the designed keyframe extraction model outperformed all the other methods, achieving an accuracy rate of 0.996, a recall rate of 0.984, and an F1 score of 0.936 on the dataset used in the study. This model’s average keyframe redundancy was 0.02, and the missed and false detection rates were both below 0.25. This indicated a strong ability to recognize key content in videos. Meanwhile, the model’s performance changed little under the test with the addition of random noise perturbation, demonstrating good robustness and generalization ability. The detection error converged to the minimum value of 0.126, and the highest value of prediction box generation accuracy could reach 0.834, which was 41.57% improved. In the video processing of intangible cultural heritage, the missing rate and false positive rate of the target object were at the lowest level as low as 0.20. Through keyframe extraction and target detection, the study realizes the effective protection and analysis of intangible cultural heritage cultural videos, and promotes the inheritance and dissemination of intangible cultural heritage.

## 1. Introduction

Intangible cultural heritage (ICH) is a variety of practices, performance forms, skills, and their related tools and artifacts continuously created by human beings in their interaction with nature and history. ICH is an important manifestation of the diversity of traditional Chinese culture. It carries the group historical memory and national spirit of the nation and provides resources for traditional cultural education and contemporary cultural innovation [1–2]. Video is a form of ICH dissemination and protection. It can simultaneously record and disseminate visual and auditory elements of ICH, providing a convenient and efficient way to share it. Meanwhile, video has an irreplaceable position in the protection process of ICH. The original form and performance style of NRLs can be preserved through video recording, providing real historical witnesses for future generations [3–4]. The finished video products related to ICH contain a large number of directly recorded long videos. Their content is rich and informative, which can comprehensively reflect all aspects of NRH programs. For the purpose of subsequent access, research, and protection, long videos are usually segmented into short videos based on their semantic content. The representative, pedagogical, and dissemination-value segments are extracted from the long videos [5]. In addition, keyframes are the most informative frames in ICH videos, which contain the core and important information of the videos. Moreover, keyframes have a strong visual impact and convey emotion effectively. Keyframe extraction can facilitate the quick presentation of non-legacy videos and aid in spreading and popularizing non-legacy culture during the retrieval, preservation, and distribution of these videos. However, at present, the segmentation and keyframe extraction of long videos mostly rely on manual operation, and the accuracy of scene segmentation and key content extraction is poor. The inability to accurately and quickly extract the key content and complete the segmentation of the video has become a major challenge hindering the protection of ICH videos [6].

In view of this, to realize the protection and analysis of long videos of ICH, and to improve the video content extraction efficiency and accuracy, the study firstly innovatively designs a keyframe extraction model based on shot boundary detection based on modern image processing and video analysis technology. Then based on the extracted video keyframes, the study improves the anchor free box detection (AFBD) algorithm. A target detection algorithm based on adaptive weight assignment is designed by introducing feature pyramid network (FPN) and attention mechanism to locate specific targets in the video to complete the segmentation processing and analysis of the video. The study applies the keyframe extraction and adaptive weight assignment methods to the preservation and analysis of ICH videos. This can enrich the application scenarios of image processing and video analysis technology and promote the continuous development of image processing technology.

This study is innovated in the following three aspects: (1) It introduces a 3D ResNet-18-based keyframe extraction model that uses spatio-temporal convolution to detect semantic changes between video frames. Because it does not rely on static frame templates, the model enhances the understanding of unstructured video content. (2) Ratio-Net, an anchor-free object detection algorithm, is developed and integrated with an attention mechanism. This improves spatial region perception and enhances recognition of complex targets, eliminating the need for manually defined anchor boxes. Third, the study seamlessly integrates keyframe extraction and object detection into an optimized, unified framework for ICH videos. This addresses the current lack of domain-specific video analysis techniques in this field.

This study is contributed by the following three aspects: (1) It proposes an automatic analysis framework tailored for ICH video processing that effectively addresses practical challenges, such as high content redundancy, low processing efficiency, and difficulty identifying meaningful segments. (2) It improves the semantic understanding of video structures by optimizing the processes of keyframe extraction and object detection, thereby enhancing the accuracy and automation of content segmentation. (3) It offers a practical and scalable solution for archival management, structured indexing, and the visual presentation of ICH video resources. It demonstrates strong adaptability and broad potential for the preservation of digital heritage.

In total, there are four sections to the research. The first section summarizes the current state of research related to video keyframe and target detection. The second section designs a video keyframe extraction model based on shot boundary detection and a target detection algorithm based on adaptive weight assignment. In the third section, the performance test and application analysis of the target detection algorithm and keyframe extraction model are carried out. The fourth section outlines the main conclusions of the experiments and future work.

## 2. Related works

Keyframe extraction is an important part of video data processing. Numerous academics have conducted in-depth study on keyframe extraction technology, which is still developing due to the ongoing advancements in deep learning (DL) and other technologies. A keyframe detection framework for action analysis of badminton players was developed by Sarwar M. A. et al. The system used 3D:VIBE posture estimation to retrieve skeleton data after first dividing macro activity into micro activity. Then, keyframes of each phase were identified and the activities were recognized by the pose and motion detection module [7]. To address fast channel switching and packet loss repair in low-latency live video distribution while maintaining steady-state viewing performance, Mareen M minimized the impact on normal video streams through keyframe injection techniques and evaluated the implementation requirements and quality impact under three coding standards. The outcomes indicated that the quality degradation caused by keyframe injection was usually low, but in rare cases drift error artifacts were present. It was recommended that the codecs disabled submotion estimation or optimized filter design [8]. To explore the efficacy of combining convolutional neural network (CNN) and recurrent neural network (RNN) in video classification and the impact of the utilization of temporal information on the performance of video classification, Savran Kızıltepe R et al. innovatively proposed the keyframe extraction strategy based on action templates by using transfer learning to realize the combination of different neural networks. According to experimental findings, this technique greatly increased the video classification accuracy [9]. Xu T. et al. proposed a visual synchronous localization and mapping algorithm based on time-delay feature regression and keyframe position optimization. This algorithm addressed issues such as image blurring, keyframe loss, and drift in trajectory construction when working with robots at large and fast view angles. The study utilized a multi-scale RNN to correct image deformation and motion blur, and introduced grayscale motion processing to supplement the keyframes. Meanwhile, the camera attitude and trajectory were optimized by combining time-delay feature regression with dual measurement constraints. Experimental results revealed that the method improved the localization accuracy by more than 10% compared with the traditional algorithm in fast and large viewing angle scenes [10]. Lin X et al. suggested a keyframe-based continuous direct sparse visual ranging algorithm to increase the precision and resilience of a real-time depth image simultaneous localization and map generation system in the presence of low image quality. The method incorporated an intrinsic keyframe selection mechanism to reduce tracking error. It supplemented this mechanism with directional acceleration segment test features and rotationally independent basic feature identification and subsequent optimization. Experimental results revealed that the system outperformed existing methods in public benchmark tests, and was particularly robust in challenging scenarios such as fast motion, texture missing, and strong lighting [11]. To optimize the recognition and summarization techniques for important activities in surveillance videos and to improve the efficiency of video processing, Pandian A A et al. proposed a multilevel clustering keyframe selection algorithm. The method reinforced inter-activity correlation, facilitated effective activity integration, and could generate efficient and informative video summaries [12]. Liu X et al. suggested a high-quality frame extraction technique that combines baseline constraints with fuzzy frame removal to improve the fidelity of video measurements. These keyframes were also used to recreate the 3D model. Experiments demonstrated that the method was robust in airborne triangulation, improved the reconstruction accuracy by more than 0.2 mm, and effectively optimized the reconstruction effect in different video scenes [13].

In a similar vein, researchers from a wide range of disciplines are concentrating on target identification algorithms, which are one of the key areas of study in computer vision. To enhance the detection speed and accuracy of on-board sensing technology for self-driving cars, Wang H et al. proposed you only look once version 8-QSD (YOLOv8-QSD) network. The network adopted a structural reparameterization technique to optimize the backbone, combined multi-scale feature fusion with bidirectional FPN, and introduced a novel query pipeline structure to cope with the remote detection challenge [14]. Chen M et al. introduced the Weather-OD weather-aware target detection approach to improve the effectiveness of target identification techniques in marine surveillance systems during severe weather conditions. It combined on-board edge and onshore cloud systems, dynamically selected machine learning models to adapt to weather changes, and ensured high-precision and low-latency detection. Experiments demonstrated that the method significantly improved the average accuracy of maritime target detection under rainy and foggy conditions, and could be continuously optimized by retraining with small datasets [15]. To enhance the performance of 3D target detection in complex environments, especially for small targets and challenging conditions, Wang C H et al. proposed the voxel-pixel fusion network VoPiFNet. This study innovatively designed a voxel-pixel fusion layer, fused LiDAR voxel, and image pixel features through a cross-modal attention mechanism, and introduced a four-parameter enhancement of fusion effect. Tests on the dataset indicated that the method outperformed the most advanced methods in challenging category detection, with an excellent overall 3D average accuracy performance [16]. For enhancing the spatial location accuracy of road sign objects in GIS using exchangeable image file data, Taşyürek M et al. proposed a hybrid method. This study combined target detection, distance estimation, rotation and projection techniques to detect road signs in EXIF and compute their spatial locations through a DL model. The outcomes revealed that the method outperformed the traditional method in terms of F1 score and location accuracy, and provided an effective means for GIS spatial data updating [17].

In summary, keyframe extraction and object detection methods have had some success in certain scenarios, but they still have limitations. First, approaches such as keyframe extraction and action template methods typically depend on scene priors, rendering them unsuitable for ICH videos, which tend to be loosely structured and semantically complex. Second, object detection models like YOLOv8-QSD depend on anchor-based mechanisms, which require the manual configuration of different sizes and aspect ratios. This restricts the model’s flexibility and its ability to generalize across different domains. Lastly, although 3D detection networks like VoPiFNet incorporate voxel-pixel fusion mechanisms, these networks are primarily intended for LiDAR data. Consequently, these networks exhibit modeling bias when applied to RGB videos and lack the ability to capture temporal features.

To overcome these limitations, this study introduces a 3D ResNet-18 structure based on shot boundaries to extract keyframes. This structure uses spatio-temporal convolutions to capture dynamic semantic transitions between video frames. This approach eliminates the need for static inter-frame features or predefined templates, thereby improving the analysis of unstructured video content. The study proposes Ratio-Net, an anchor-free object detection algorithm that incorporates an attention mechanism to improve spatial region perception, for object detection. Unlike anchor-based structures, such as YOLOv8, the proposed method eliminates anchor design constraints and enhances the accuracy and stability of recognizing irregular and low-texture targets in ICH videos. Additionally, this framework unifies optimization from video structure parsing to semantic recognition by integrating the keyframe extraction and object detection processes. This effectively addresses the current issues of fragmented workflows and weak robustness in cultural video processing.

## 3. ICH video protection and analysis study

The study initially develops a keyframe extraction algorithm based on the concept of shot boundary detection in an attempt to safeguard and analyze ICH films and facilitate the inheritance and spread of ICH. Then, an improved target detection algorithm based on adaptive weight assignment is designed by taking keyframes as input.

### 3.1. Ethical and cultural sensitivity statement

All of the video data related to ICH used in this study are obtained from publicly available datasets (e.g., ClipShots, TRECVID, and RAI). These datasets adhere to platform usage regulations and data-sharing permissions. Throughout the development and experimentation with the model, the research team has maintained a high level of respect for cultural sensitivity and intellectual property. All ICH content included in the study is publicly available and does not include unauthorized personal or community-specific footage. For future studies involving specific communities or cultural subjects, the research team will adhere strictly to ethical standards regarding informed consent, data protection, and cultural preservation. This ensures that all research activities are lawful, compliant, and respectful of cultural rights.

### 3.2. Dataset statement

The collection and analysis method complied with the terms and conditions for the source of the data.

### 3.3. Keyframe extraction for ich videos based on shot boundary detection

**Fig 1:**
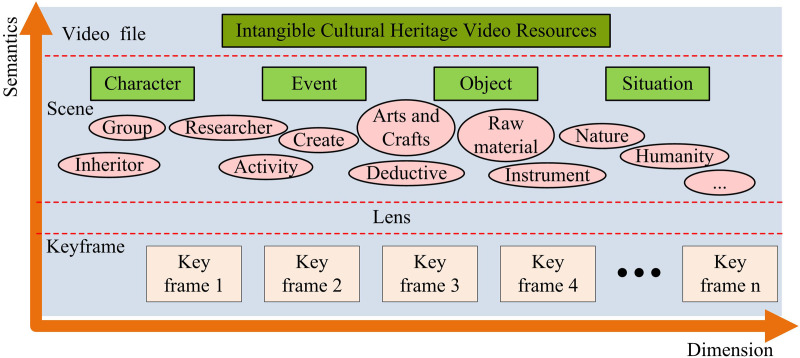
Schematic diagram of the video structure composition of intangible cultural heritage content.

To display the unique charm and value of ICH programs in all aspects, the content of long videos recording ICH usually covers the introduction of the inheritors, the process of techniques and representative works, and the videos are rich in content and long in duration. The lengthy ICH videos exhibit enormous amounts of information and intricate details, making video processing more complex and preservation more challenging. Therefore, extracting representative keyframes from ICH long videos can significantly reduce the amount of data. Further target detection based on keyframes can complete the classification, labeling, storage of the video, and promote the protection and inheritance of ICH. In conclusion, one crucial way to achieve the digital protection and distribution of ICH video content is to divide long videos into shorter ones. To achieve effective protection and management of ICH videos, keyframe extraction and target detection are required to complete the video segmentation process [18]. In this regard, keyframe extraction research is firstly carried out for ICH long videos. The structure of ICH content-related video is shown in Fig 1.

In Fig 1, the ICH video is divided into four levels: file, scene, shot, and keyframe. Frame is the smallest unit of video. Based on the shot boundary detection, the study develops keyframe extraction, which splits the video into different shot segments, so as to extract keyframes in each shot segment. Meanwhile, the study divides the shot boundary detection task into two parts: shot boundary localization and cut edge shot detection. However, to lessen the interference of redundant information, the region without a shot boundary must be filtered first due to the high volume features of ICH video. The expression of the change process between different shots is expressed in Equation (1).


$$I_{t}(x)=\alpha_{t}(x)B_{t}(x)+(1-\alpha_{t}(x))F_{t}(x)$$ (1)

In Equation (1), α represents the mixing coefficient, which controls the intensity with which the current frame is discriminated as the boundary of the shot. The larger the α, the more inclined the model is to identify abrupt boundaries, making it suitable for scission-type shots. As α approaches smaller, it becomes more sensitive to gradual transition type shot switching. $I_{t}(x)$ denotes the pixel value of the image at position x at time t moment. $B_{t}$ and $F_{t}$ denote the pixel values of the first shot and the second shot, respectively.

**Fig 2:**
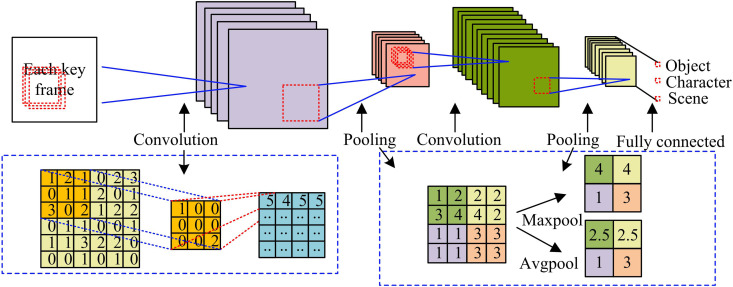
Working mechanism of shot boundary localization network.

The research uses a CNN to extract features from different video frames. More specifically, the output of the fourth residual block of the ResNet-18 network (i.e., the conv4 layer) is used for feature extraction. The output feature map (FM) of this layer has dimensions of 7 × 7 × 256. Calculating the L2 norm difference of FMs extracted from adjacent frames at this layer effectively measures the degree of semantic change between frames. This achieves preliminary detection of the no-shot boundary area. The basic structure of CNN usually consists of an input layer, a convolutional layer (CL), a pooling layer (PL), a fully connected layer (FCL), and an output layer. The working mechanism is shown in Fig 2 [19–20].

In Fig 2, the CNN extracts local features of the data through a series of learnable filters. The convolution operation captures low-level features (LLFs) such as edges, corner points, textures, etc. in the image by performing element-by-element multiplication and then summing with the input data through a moving convolution window. The PL then downsamples the FM as a way to reduce the amount of computation and number of parameters. This increases the network’s translational invariance to the input data and usually contains both maximum and average pooling operations [21–22]. The final fully-connected layer then performs a global analysis of the features extracted from the CL and PLs, maps the features to the output space, and produces the final classification results. The convolutional computation process is shown in Equation (2).


$$x_{j}^{l}=f\left(\sum_{i\in M_{j}}x_{i}^{l-1}*W_{ij}^{l}+b_{j}^{l}\right)$$ (2)

In Equation (2), f denotes the activation function. $W_{ij}^{l}$ denotes the filter weight at position (i,j) of layer l. $b_{j}^{l}$ is the bias. x denotes the FM. $M_{j}$ denotes FM. The spatial feature F of all frames of the video is extracted using CNN network and the difference degree $d_{i}$ between different neighboring frames is calculated as shown in Equation (3).


$$d_{i}=1-\frac{\left\langle F_{i},F_{i+1}\right\rangle }{\left\|F_{i}\right\|\left\|F_{i+1}\right\|}$$ (3)

After obtaining all neighboring frames $d_{i}$ according to Equation (3), the average value μ of $d_{i}$ is calculated. The decision condition of the final candidate boundary frame is shown in Equation (4).


$$(d_{i}>\lambda d_{i-1}\cup d_{i}>\lambda d_{i+1})\cap d_{i}>\gamma \mu$$ (4)

In Equation (4), λ is the magnification ratio for controlling the local difference degree. γ is the relative threshold for controlling the difference from the global average. Together, they determine the shot boundary. The values are 1.2 and 0.25, which correspond to local mutation sensitivity and global difference tolerance, respectively. These values are obtained through the grid tone parameters of the training set and effectively balance the boundary recognition performance of shear and gradient shots.

After the initial localization of the shot boundary, the video cutover shot needs to be detected. First, the potential location of the shot switch in the video is confirmed. Candidate observations are then performed within a few frames before and after the suspicious location to more accurately determine the switching point. Then, based on the candidate observations, further analysis is performed to distinguish between tangential and gradual shot switching. A cut is a direct, abrupt switch between two shots. The keyframe boundary of a cutaway shot is the frame with the largest difference between frames. A fade is a smooth transition between two shots through a transition effect. The keyframe boundary of a fading shot then needs to be determined according to the change process of the beginning and ending frames of the fading [23–24]. The study takes the candidate boundary frame x as the center, and takes [x−5,x+10] as the sampling time range to extract the continuous frame segments as the input of the model.

**Fig 3:**
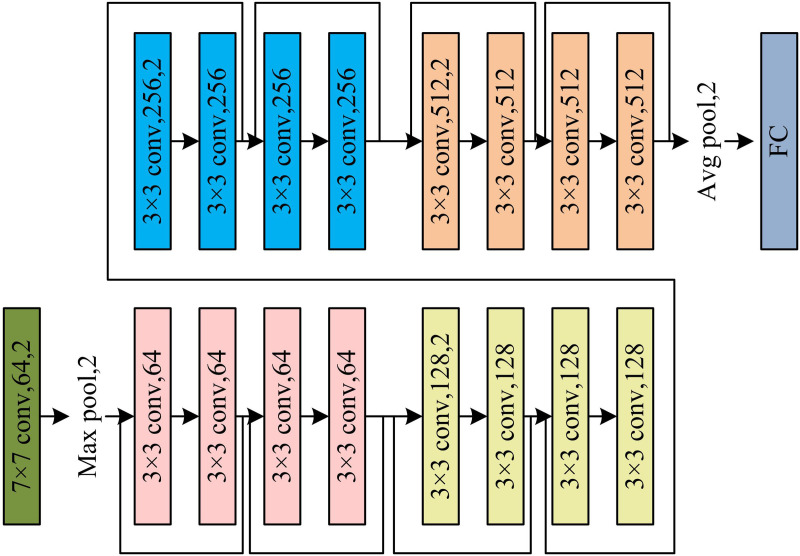
Schematic diagram of ResNet-18 structure composition.

To accurately obtain the temporal and spatial position information of the switching shots and the fade-in and fade-out shots, 3D ResNet-18 is selected as the backbone network for keyframe extraction in the study. ICH videos are usually loose in structure, slow in rhythm and sparse in semantics. The 3D ResNet-18 model effectively captures action changes and scene transitions without increasing excessive computational overhead through residual connections, which achieve robust extraction of spatio-temporal features. Compared to lightweight networks such as MobileNetV3 and EfficientNet, ResNet-18 strikes a better balance between feature expression ability and training stability. It is well-suited to the in-depth analysis requirements of cultural videos. The form of its network structure is shown in Fig 3.

In Fig 3, the ResNet-18 network mainly consists of 7 × 7, 3 × 3 CLs, FCLs, constituting 8 residual blocks and 4 residual layers. Compared with the 2D ResNet-18, the size of the convolutional kernel (CK) of the 3D ResNet-18 is increased by a dimension that takes into account the “continuous frame” parameter. The residual structure connects the different 3DConv modules to efficiently extract temporal information from the input as well as from the FMs, helping the network to process the 3D data of the video [25–26]. The computation process of the residual block for the L th of 3D ResNet-18 is shown in Equation (5).


$$X_{L}=X_{l}+\sum_{i=l}^{L-1}F(X_{i},W_{i})$$ (5)

In Equation (5), F denotes the mapping function of input and output. $X_{l}$ and $X_{L}$ denote the input and output of the previous layer. $W_{i}$ denotes the weight. The residual block is added directly from the input to the output of the residual block through jump connections, which allows the network to maintain a good performance while the depth is increased. The 7 × 7 CL in 3D ResNet-18 is mainly used to extract LLFs from the input image. The 3 × 3 CL is mainly used to further reduce the size of the FM. 3D ResNet-18 uses batch normalization (BN) technique to improve the stability of the CK output [27]. 3D ResNet-18 converts the FMs into feature vectors through the global average pooling (GAP) layer and classifies them with the help of a FCL, as shown in Equation (6).


$$x_{k}^{\left(m\right)}=f(\beta_{k}^{\left(l\right)}down(x_{k}^{\left(l-i\right)})+b_{k}^{\left(l\right)})$$ (6)

In Equation (6), down denotes the downsampling operation in the PL. β denotes the downsampling multiplier. k denotes the number of CKs. Finally, in order to ensure the DetA of the video cut shot, the study launched a post-processing operation on the prediction results, which calculates the HSV color histograms $H_{x}$, $H_{x+1}$, and barrage distance $d(H_{x},H_{x+1})$ of the adjacent prediction frames of the cut shot, respectively. The calculation process is shown in Equation (7).


$$d(H_{x},H_{x+1})=1-\frac{1}{\sqrt{H_{x}H_{x+1}}}\sum \sqrt{H_{x}(i)H_{x+1}(i)}$$ (7)

Compare the barotropic distance $d(H_{x},H_{x+1})$ with the set threshold. When $d(H_{x},H_{x+1})$ is less than the threshold, no shear has occurred at the boundary of this candidate shot. For this study, the training parameters of the 3D ResNet-18 model are conFigd as follows: the length of the input video clip is set to 16 frames, and the stride is set to 1. Moreover, Pre-trained weights from the Kinetics-400 dataset are used for transfer learning. The Adam optimizer is employed with an initial learning rate of 1e-4, and a cosine annealing schedule is adopted for dynamic adjustment. The model is trained for 50 epochs. Data augmentation techniques, such as random cropping, horizontal flipping, and brightness perturbation, are applied during training to enhance generalization.

### 3.4. Target detection algorithm based on adaptive weight assignment

**Fig 4:**
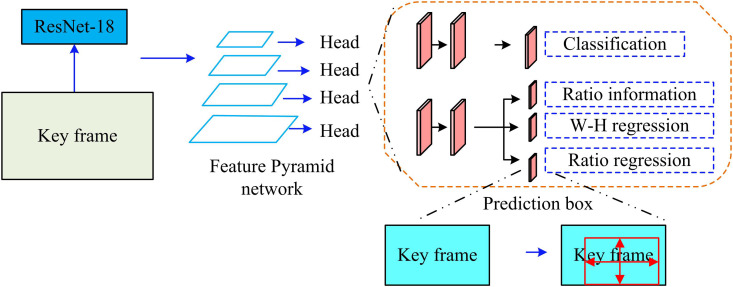
Schematic diagram of anchor free box detection algorithm framework based on ratio information.

After extracting the video keyframes based on shot boundary detection, the study unfolds the target detection analysis based on the keyframes, which facilitates the labeling and categorization of ICH videos. The study selects AFBD algorithm as the basic technology of ICH target detection. Furthermore, instead of setting a predefined anchor box to make target detection easier, it forecasts the target object’s width, height, center point coordinates, and category likelihood directly. The traditional AFBD mainly consists of keypoint-based and center-based detection algorithms. However, keypoint-based target detection is not very good at perceiving the internal information of the target object. Meanwhile, the accuracy of keypoint pairing is low due to the limitations of corner point information. Additionally, the detection algorithm that predicts the center point easily ignores global information and has poor adaptability to objects of special shapes [28–29]. In this regard, a ratio information network (Ratio-Net) is proposed in the study. Fig 4 displays the framework for the algorithm.

In Fig 4, the Ratio-Net target detection algorithm consists of backbone network ResNet-18, classification branch, and regression branch. Meanwhile, Ratio-Net unfolds the detection of target objects of different sizes with the help of FPN. The core idea of FPN is to detect and recognize targets at different scales, and to solve the detection and segmentation of objects at different scales by constructing multi-scale FPN. Ratio-Net first extracts FMs at different scales from the keyframe images through the base network ResNet-18. Rich feature representations at various scales are then obtained by fusing the LLF maps with the high-level FMs.

Each branch of Ratio-Net has the following structure: the classification branch consists of three 3 × 3 CLs and one 1 × 1 output CL. All of these layers use the ReLU activation function. The bounding box regression branch has a similar structure, but its output channels are adjusted to 4 × K. Among them, K is the number of categories, each of which outputs a set of box parameters (center coordinates, width, and height). The ratio information branch consists of two 3 × 3 CLs, followed by a Sigmoid normalization layer. This layer outputs the relative width and height ratios of the target. The Sigmoid function restricts the output to the range [0, 1] and multiplies it by the dimensions of the bounding box. This refines the boundaries and enhances the model’s adaptability to targets of irregular shape.

**Fig 5:**
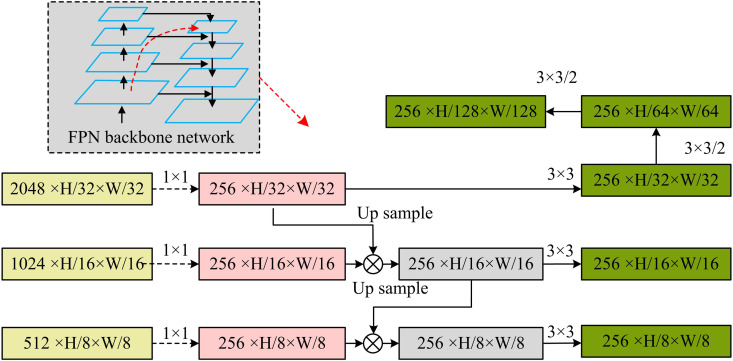
Schematic diagram of FPN network structure.

Fig 5 displays the FPN network structure that is built during the investigation.

In Fig 5, the FPN interpolates the higher-level FMs using an upsampling operation to obtain FMs matching the size of the corresponding lower-level FMs. Then, feature fusion is accomplished by element-by-element summation for subsequent classification and regression operations [30–31]. Furthermore, FPNS outputs at different layers consist of three branches: classification, regression, and ratio information. The loss weights for these branches are 0.4, 0.4, and 0.2, respectively. The classification and regression branches play a central role in the detection task, so they are assigned higher weights. The ratio information branch is primarily used to refine the size of the bounding box. To avoid its unstable impact on the overall optimization process, the weight is appropriately reduced in order to balance its auxiliary role with the stability of the overall training process. Moreover, all points in the rectangular box are considered as positive samples in the classification branch task. Equation (8) displays the training loss function $L_{cls}$ ‘s expression.


$$L_{cls}=\frac{-1}{N}\sum_{i=1}^{H}\sum_{j=1}^{W}\sum_{c=1}^{C}\{\begin{array}{cc} {}*20c\theta {\left(1-P_{ijc}\right)}^{\eta }\log\left(P_{ijc}\right), & ifY_{ijc}=1\\ (1-\theta )P_{ijc}^{\eta }\log\left(1-P_{ijc}\right), & otherwise \end{array}$$ (8)

**Fig 6:**
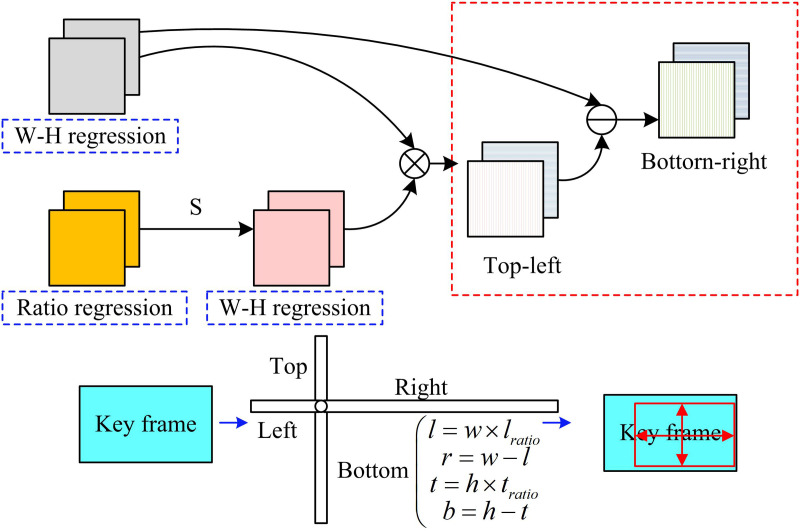
Schematic diagram of prediction box generation process.

In Equation (8), H, W, and C display the height, width, and quantity of channels of the keyframe image. θ and C denote the hyperparameters. $P_{ijc}$ is the prediction score of the category. $Y_{ijc}$ is the actual category. N is the quantity of rectangular frames in the real sample. The W – H regression in the regression branch mainly predicts the height and width of the target object, and the ratio regression (RR) mainly predicts the ratio information. The RR branch outputs the width-to-height ratio factors, which are then normalized using the Sigmoid function to constrain their values to the range [0, 1]. These ratios are multiplied by the predicted bounding box dimensions from the backbone network to determine the final box size. This improves the model’s ability to adapt to irregularly shaped objects and prevents unstable predictions caused by unbounded regression values. The generation process is shown in Fig 6.

In Fig 6, the RR is processed by the Sigmoid function and multiplied with the W – H regression to obtain the distance values from the feature points to the boundaries. The distances l, r, t, and b of the feature points of the target object to the different boundaries of the prediction box left, right, top, and bottom are calculated from the prediction box width w, prediction box height h, which are related to the ratio information. The calculation process of prediction box $P_{ij}$ generation is shown in Equation (9).


$$(\begin{array}{c} {}*20cl=w\times l_{ratio}\\ r=w-l\\ t=h\times t_{ratio}\\ b=h-t \end{array}$$ (9)

In Equation (9), $l_{ratio}$ denotes the ratio of l to width w. $t_{ratio}$ denotes the ratio of t to the height h. It should be mentioned that the prediction box’s accuracy is increased by taking into account the target object’s global information during its creation. The calculation of the training loss function $L_{reg}$ is shown in Equation (10).


$$L_{reg}=\frac{-1}{N}\sum_{i=1}^{H}\sum_{j=1}^{W}\log(IoU(P_{ij},Y_{ij}))$$ (10)

**Fig 7:**
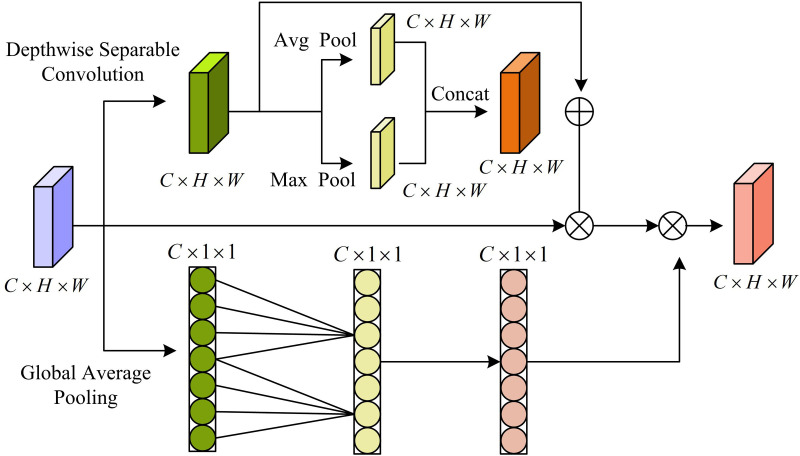
Schematic diagram of attention mechanism module framework structure.

In Equation (10), $Y_{ij}$ denotes the real frame. IoU denotes intersection over union (IoU). In summary, the generation of the prediction box depends on the ratio of information contained within the box. Points with more visual features can generate more accurate prediction boxes. To learn the important region with the largest weight in the process of prediction box generation, the study introduces an attention mechanism to construct an adaptive weight assignment for target detection. The study adds the attention mechanism module to the ratio information branch. Fig 7 displays the framework’s schematic diagram.

In Fig 7, the modular framework of the attention mechanism designed by the study mainly consists of depthwise separable convolution (DSC) and GAP. DSC is an efficient convolution operation that consists of two separate processes, depth convolution and point-by-point convolution. The deep convolution (DC) method reduces the quantity of model parameters by performing the convolution operation using a different CK for each channel of the input feature image. Furthermore, a maximum PL and an average PL are the results of DC, respectively. The data’s spatial dimension is decreased by the maximum PL, which chooses the highest value from each local region of the input FM. To maintain the image’s background information, the average PL, on the other hand, determines the average value of each local region of the input FM. The output of DC is processed by DSC using a 1 × 1 CK during point-by-point convolution. It achieves the fusion of information between channels and modifies the output FM’s channel count. The study uses the Sigmoid function to limit the position interval of the output FM and the expression is shown in Equation (11).


$$S\left(x\right)=\frac{1}{1+e^{-x}}$$ (11)

Finally, the GAP module performs an average pooling operation for each channel of the FM to map the high-dimensional data into one-dimensional vectors. The output $\mu_{c}$ is computed in Equation (12).


$$\mu_{c}=\frac{1}{H\times W}\sum_{h=1}^{H}\sum_{w=1}^{W}f_{c}(h,w)$$ (12)

In Equation (12), $f_{c}(h,w)$ denotes the c th channel in the FM. The lack of extra parameters needed for GAP, as opposed to the conventional FCL, lowers the model’s complexity and overfitting risk.

## 4. Performance testing and application effect analysis of video keyframe extraction and target detection

The study conducts model performance testing and application analysis in an effort to validate the effectiveness of the research-designed keyframe extraction model and target detection algorithm in ICH video protection and analysis.

### 4.1. Performance testing of keyframe extraction and target detection algorithms

The study starts with the performance testing of the algorithm. The Windows 11 operating system is used for the trials. The central processor is Intel Core i5-12500 with a processor frequency of 3 GHz. The image processor is NVIDIA GeForce RTX 2060. The programming language is Python 3.8.8 and the DL framework is PyTorch 1.12.1. ClipShots, TRECVID, and RAI are selected as the experimental datasets. The ClipShots dataset contains short videos of more than 20 categories collected from YouTube and Weibo for shot boundary detection. TRECVID contains annotated videos related to shot boundary detection, which can be used for video analysis and processing. RAI also provides a large amount of labeled shot boundary information for tasks such as video shot segmentation and retrieval. To unify the experimental process and better align with the requirements of ICH-related video tasks, standardization is applied to some original labels in the ClipShots, TRECVID, and RAI datasets. During this process, all shot boundary annotations are converted into frame-level start and end indices using a consistent timestamp format based on frame counts. Ambiguous or inconsistent gradual transition labels are reclassified into clearly defined categories, such as “fade-in,” “fade-out,” or “dissolve.” Additionally, samples with obvious labeling errors or missing annotations are corrected or excluded as appropriate. The experimental data are divided into the training set and the test set in an 8:2 ratio. The comparison models include the action template-based keyframe extraction strategy from literature [9], the multilevel clustering keyframe selection algorithm from literature [12], and the high-quality frame extraction method combining fuzzy frame removal and baseline constraints from literature [13].

Prior to the comparison experiments, hyperparameter ablation experiments are first performed to validate the hyperparameter λ and the lens boundaries and to determine the effect of Equation (4) γ on the keyframe extraction performance (accuracy and redundancy). The results in Table 1 show that when λ and γ are in a reasonable range. Moreover, the overall performance of the model is stable. Among them, the best extraction performance is achieved when λ =1.2 and γ =0.5, with an accuracy of 0.991 and a redundancy of only 0.021. In contrast, when λ is smaller (such as 1.0), the model is less sensitive to boundary changes. Furthermore, missing some boundaries results in decreased accuracy. When γ is smaller (such as 0.15), the model is too sensitive to non-boundary areas, with more misjudgments and a significant increase in redundancy. Conversely, when γ is too large, such as 0.30, the model tolerance is too high. This results in some weak but real shot transitions being missed, which slightly decreases accuracy.

**Table 1:** Results of hyperparameter ablation experiments.

λ	γ	Accuracy	Redundancy
1.0	0.20	0.982	0.045
1.2	0.25	0.991	0.021
1.4	0.30	0.984	0.023
1.2	0.15	0.993	0.038

**Fig 8:**
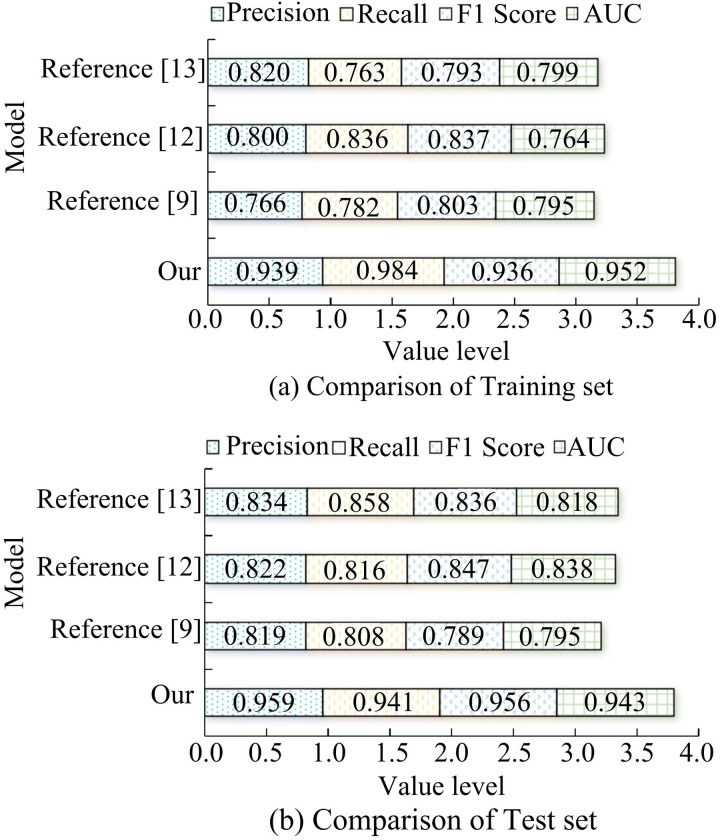
Performance comparison of different keyframe extraction models.

Fig 8 displays the outcomes for various keyframe extraction models in terms of precision, F1 score, recall, and area under the curve (AUC) of the receiver operating characteristic curve (ROC). In Fig 8(a), the keyframe extraction model designed for the study achieves 0.939 precision, 0.984 recall, 0.936 F1 score, and 0.952 AUC on the training set. In the same experimental setting, the other three models have the highest precision rate of 0.820, the highest recall rate of 0.836, and the highest F1 score of 0.837. The same model has a poor trade-off between precision rate and recall rate. In Fig 8(b), the keyframe extraction model designed by the study also performs best in the test set. Taken together, the method covers all important frames when extracting keyframes, and the model performs poorly in distinguishing between keyframes and non-keyframes.

**Fig 9:**
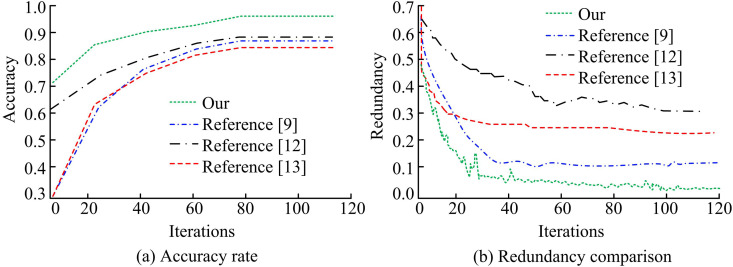
Comparison of accuracy and redundancy of different keyframe extraction models.

Fig 9 compares the redundancy and accuracy of various keyframe extraction models. In Fig 9(a), the accuracy of the keyframe extraction model designed by the study increases rapidly at the beginning of iterations. When 20 iterations are made, the accuracy approaches the maximum value of 0.996 and remains stable. The accuracy of the keyframe extraction model based on action templates rises slowly at the beginning of the iteration. The accuracy of the multilevel clustering keyframe selection algorithm with the high quality frame extraction method of literature [13] rises faster at the beginning of the iteration. However, the accuracy value is always lower than that of the research design. It can be concluded that the keyframes extracted by the model designed by the study are the most compatible with the given keyframes. However, in a few cases, the model may misjudge the boundaries if the semantic changes between consecutive shots are too subtle, or if occlusion interference occurs. This can result in redundancy or missed detection in keyframe extraction. In Fig 9(b), the redundancy of the keyframe extraction model designed by the study decreases rapidly at the beginning of the iteration and the minimum redundancy converges to 0.02. The redundancy of the other three models is significantly higher than that of the studied design. It can be observed that the keyframes extracted by the model did not contain more similar frames. The experimental results are verified by the *p*-test. The significance level *p* < 0.05, indicating that the performance improvement of the proposed method is statistically significant and the results are reliable.

**Fig 10:**
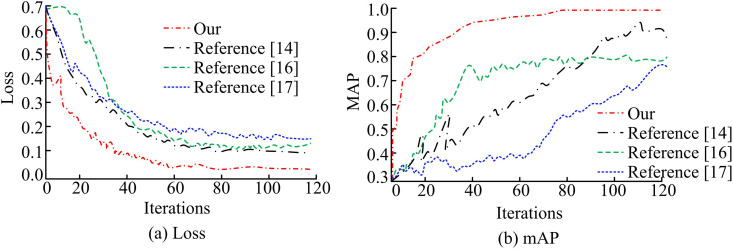
Comparison of mAP and loss function for different models.

The target detection algorithm designed by the study based on adaptive weight assignment is compared with the YOLOv8-qsd network of literature [14], the voxel-pixel fusion network VoPiFNet of literature [16], and the hybrid target detection network of literature [17]. Fig 10 displays the mean average precision (mAP) against loss function curves for several target detection algorithms. In Fig 10(a), the loss function of the target detection algorithm based on adaptive weight assignment decreases rapidly at the beginning of the iteration, showing faster convergence and lower final loss values, with a minimum loss value of 0.03. The other three models do not have superior models for loss function optimization. The target detection algorithm based on adaptive weight assignment has a stronger learning ability during the training process. In Fig 10(b), the mAP curve of the target detection algorithm based on adaptive weight assignment has the fastest convergence speed. The mAP value approaches 1.0 after about 20 iterations. The mAP curves of the other three models in the same experimental setting are less stable and have slower convergence speeds. Taken together, the design of the study achieves optimal DetA and generalization ability. The experimental results are verified by the *p*-test. The significance level *p* < 0.05, indicating that the performance improvement of the proposed method is statistically significant and the results are reliable.

**Fig 11:**
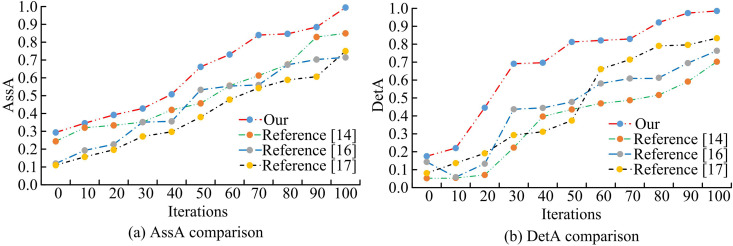
Comparison of association accuracy and detection accuracy of different models.

To further compare the video detection and analysis capabilities of different models, association accuracy (AssA) and DetA are selected as evaluation metrics. Fig 11 displays the outcomes of the experiment.. In Fig 11(a), the target detection algorithm based on adaptive weight assignment achieves the highest AssA value of 0.995. The AssA values of YOLOv8-qsd network, voxel-pixel fusion network VoPiFNet, and hybrid target detection network are 0.850, 0.715, and 0.750, respectively. Higher AssA values represent that the models can track the objects stably between different video frames, and the ability to correctly correlate the detected objects between video frames is stronger. The other models are not sensitive enough to changes in the target object. In Fig 11(b), there are significant differences among the DetA values of different models. The object detection algorithm with adaptive weight allocation achieves the highest DetA value, at 0.986. However, detection box drift or association failure may still occur in scenarios with extremely uneven lighting or severe target occlusion. The higher DetA represents that the model can still accurately recognize the target object in the video frame under the changing state. The other three models have lower DetA values and are prone to false alarms of target objects. The experimental results are verified by the *p*-test. The significance level *p* < 0.05, indicating that the performance improvement of the proposed method is statistically significant and the results are reliable.

After verifying the superiority of the proposed method, it is tested on three datasets to confirm its robustness. After conducting multiple parallel experiments, the average value of the results was taken and the standard deviation was calculated, as shown in Table 2. The proposed method demonstrates high accuracy and stability across three public datasets. F1 scores reach 95.1%, 92.7%, and 93.7%, respectively, and standard deviations remain within ±0.5. This indicates that the model can generalize well under different video contents and structures. mAP@0.5 achieves the best performance on the RAI dataset at 90.4%. Under the more rigorous mAP@[0.5:0.95] metric, ClipShots and RAI reach 71.2% and 73.1%, respectively. This further demonstrates the adaptability and robustness of the method in multi-scale object detection and complex shot boundary recognition.

**Table 2:** Absolute performance metrics and standard deviations across three runs on each dataset.

Dataset	Precision (%)	Recall (%)	F1 score (%)	mAP@0.5 (%)	mAP@[0.5:0.95] (%)
ClipShots	93.8 ± 0.7	96.5 ± 0.5	95.1 ± 0.4	89.6 ± 0.6	71.2 ± 0.9
TRECVID	91.3 ± 0.8	94.2 ± 0.6	92.7 ± 0.5	88.1 ± 0.5	69.8 ± 0.7
RAI	92.5 ± 0.6	95.0 ± 0.4	93.7 ± 0.3	90.4 ± 0.4	73.1 ± 0.8

### 4.2. Application effectiveness analysis of keyframe extraction and target detection algorithm

**Fig 12:**
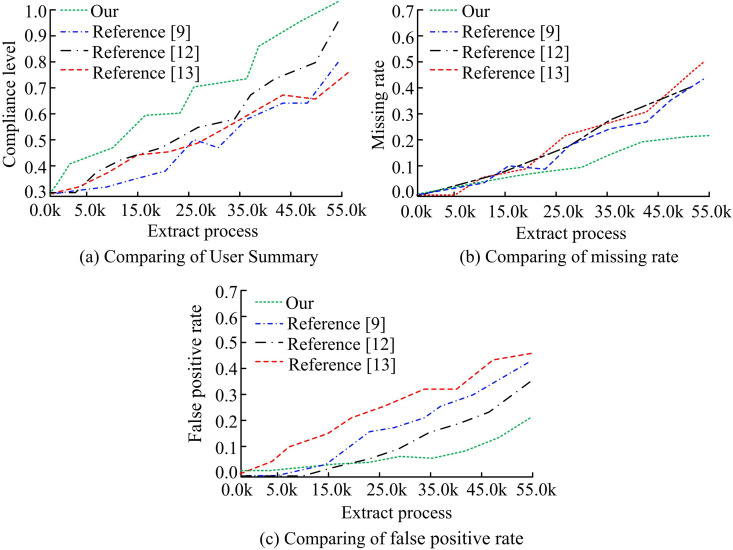
Comparison of application effects of different keyframe extraction models.

Video materials related to ICH are collected from major online platforms, research organizations, and libraries. The collected videos are preprocessed by cropping, scaling, and format conversion to ensure the consistency of the data. Meanwhile, the collected videos are manually labeled with ICH type, performance form, time and place, and background information. Moreover, frame-level annotation is performed for videos that need to be analyzed in a fine-grained manner. Subsequently, the processed dataset is used for model application analysis. The comparing of user summary (CUS), missing rate, false positive rate (FPR) of different keyframe extraction models are compared. Fig 12 displays the outcomes of the experiment. In Fig 12(a), the CUS value of the keyframe extraction model designed in the study is always at the highest level. Its extracted keyframes can accurately reflect the main content of the video, and the accuracy and consistency of the model are superior. In Fig 12(b) and 12(c), the missing rate and FPR of the research-designed keyframe extraction model are at the lowest level, taking values less than 0.25. The missing rate and FPR of the other three models are higher than 0.30. This result shows that the keyframe extraction model designed in the study successfully identifies important keyframes in videos. The model has strong recognition capabilities and does not introduce excessive noise that would affect subsequent video analysis.

**Fig 13:**
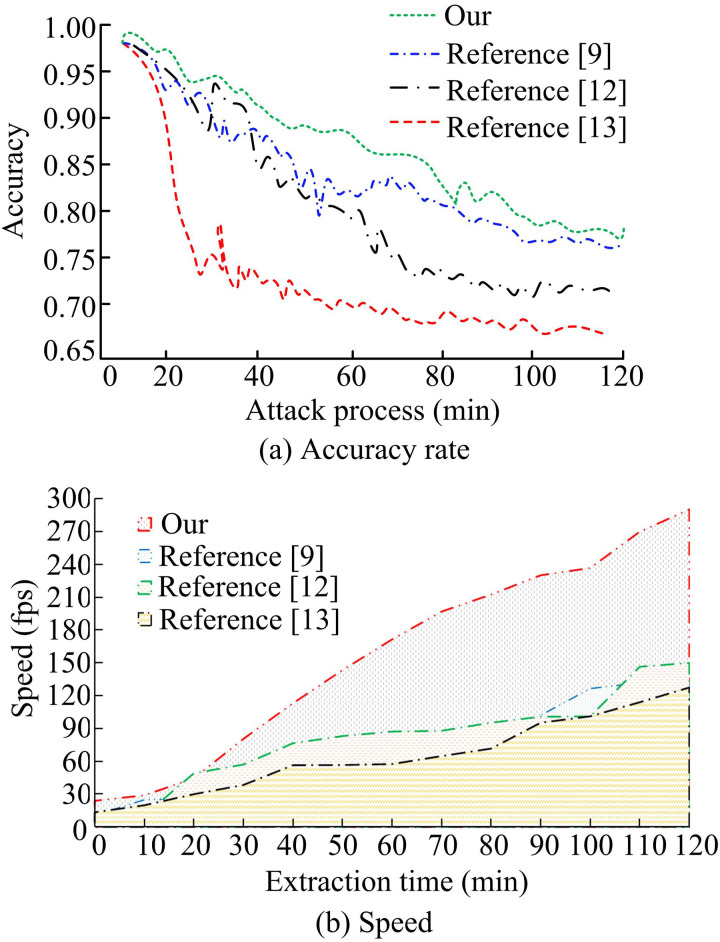
Comparison of extraction efficiency and robustness of different models.

Fig 13 compares the resilience and extraction efficiency of several keyframe extraction algorithms. In Fig 13(a), the keyframe extraction model designed by the study stays at a high level at the beginning of the introduction of noise interference attack. As the attack process proceeds, the accuracy slightly decreases, but overall stays above 0.80. In contrast, the accuracy of the other three models decreases more significantly, only the research design still shows better robustness in the face of attacks and interference. In Fig 13(b), the keyframe extraction model of the research design is the most efficient. Its extraction speed is close to 300fps in the whole extraction time range. This result shows the high performance advantage of this method when dealing with a large amount of video data. The experimental results are verified by the *p*-test. The significance level *p* < 0.05, indicating that the performance improvement of the proposed method is statistically significant and the results are reliable.

**Fig 14:**
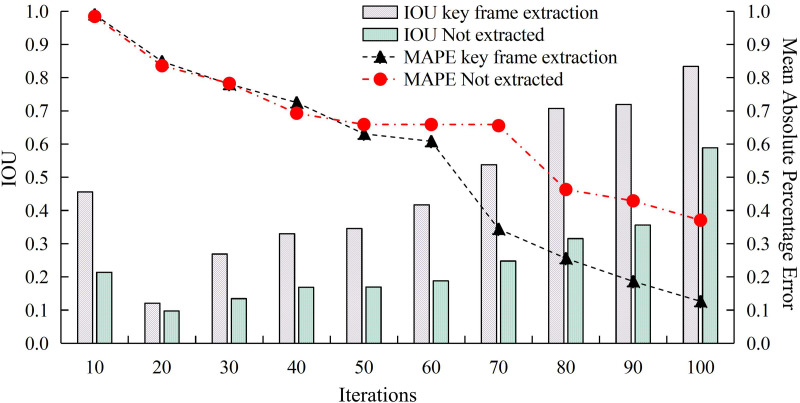
Comparison of MAPE and IOU of different models.

The effect of keyframe extraction on the target detection model mean absolute percentage error (MAPE) versus IoU is compared during ICH video processing analysis. Fig 14 displays the outcomes of the experiment. As the repetitions increases, the MAPE values for both successful keyframe extraction and direct target recognition without keyframe extraction progressively drop. However, the MAPE value of target detection after keyframe extraction decreases more in the late iteration and converges to the minimum value of 0.126. Meanwhile, target detection after keyframe extraction significantly improves the IoU value. With the highest value of 0.834, the prediction box produces higher accuracy-a 41.57% improvement. The experimental results are verified by the *p*-test. The significance level *p* < 0.05, indicating that the performance improvement of the proposed method is statistically significant and the results are reliable.

**Fig 15:**
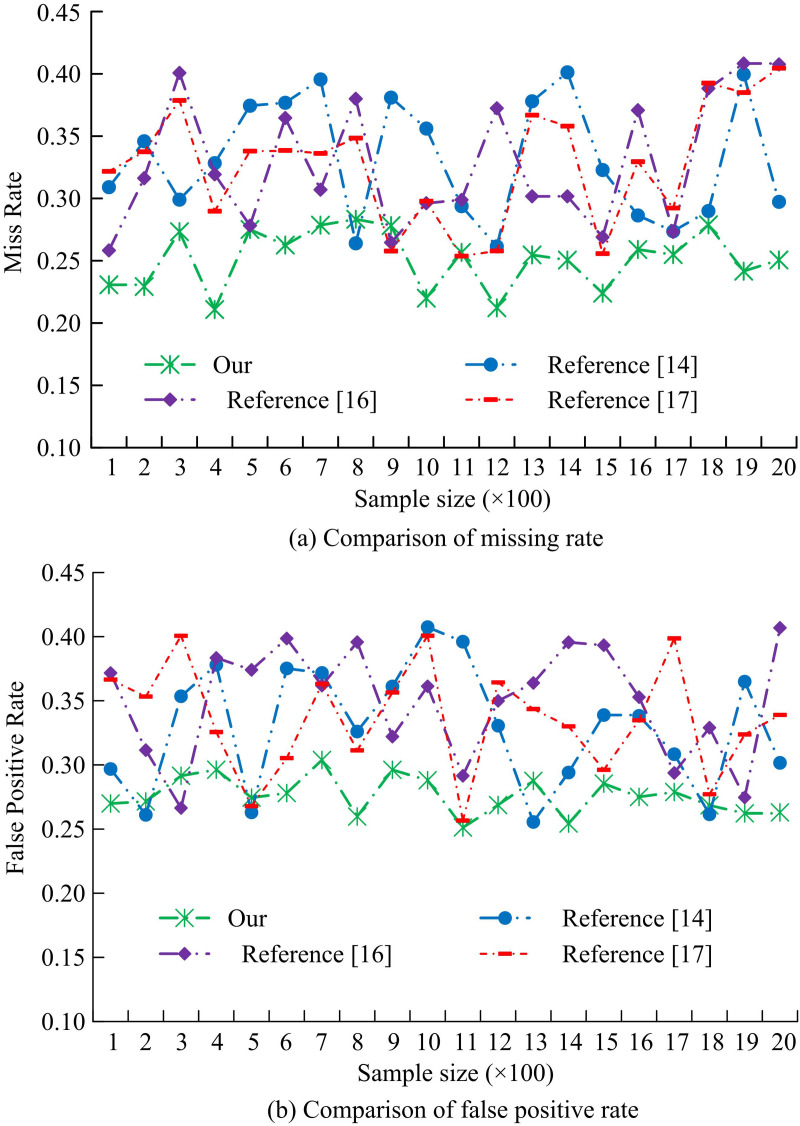
Comparison of missed detection rate and false detection rate of different object detection models.

Fig 15 compares the FPR and missing rate for several target detection methods. In Fig 15(a), the missing rate curve of the target detection algorithm based on adaptive weight assignment designed by the study fluctuates at the lowest level, with a minimum value close to 0.20. In contrast, the method fails to detect the smallest percentage of targets with the lowest missing rate. In Fig 15(b), the studied design also takes the smallest value on FPR. The model categorizes the target objects less often and handles the analysis of ICH videos better. The experimental results are verified by the *p*-test. The significance level *p* < 0.05, indicating that the performance improvement of the proposed method is statistically significant and the results are reliable.

## 5. Conclusion

This study addressed common challenges in processing ICH videos, including high content redundancy, low processing efficiency, and difficulty rapidly extracting key segments. For the cultural video context, a new automated analysis framework was proposed. This framework enhanced semantic structure understanding by jointly optimizing keyframe extraction and object detection. In the keyframe extraction module, the 3D ResNet-18 model based on shot boundary detection performed well, achieving a precision of 0.939, a recall of 0.984, an F1 score of 0.936, and an AUC of 0.952. With a redundancy rate of only 0.02, it successfully preserved essential content and reduced data volume, meeting the practical needs of ICH video archiving and management. The anchor-free algorithm with adaptive weight allocation achieved optimal performance for object detection across multiple metrics. It produced a minimum loss of 0.03, a fast and stable mAP curve, and AssA and DetA values of 0.995 and 0.986, respectively. These results indicated strong adaptability to complex backgrounds and low-texture targets. Further experiments revealed that incorporating keyframe extraction significantly improved the IoU of object detection. Accuracy of the prediction box increased by 41.57%, reflecting substantial gains in efficiency and precision. In summary, the proposed framework was a feasible and effective technical solution for the structured analysis, management of content, and visual dissemination of ICH videos. It was highly adaptable and had great potential for practical application.

## 6. Limitations and future work

Although the proposed method performs well in multiple experimental scenarios, it requires further validation to confirm its stability and adaptability in extreme lighting conditions, frequent shot transitions, and significant stylistic variations in regional ICH content. Additionally, the current model is designed for 2D video data only and does not address 3D heritage reconstruction or AR/VR-based interactive applications yet. Future research could focus on cross-domain generalization, multimodal integration (e.g., combining speech and motion cues), and adapting the framework for use in immersive environments. Studying these areas could make the framework more applicable in complex cultural contexts.

## Supporting information

S1 File Minimal Data Set Definition. (DOC)
