# Supplementary material for: Protection and Analysis of Intangible Cultural Heritage Videos Based on Keyframe Extraction and Adaptive Weight Assignment
Source: PLoS One. 2025 Aug 29;20(8):e0330176. doi: 10.1371/journal.pone.0330176 (PMC12396677; doi:10.1371/journal.pone.0330176)
Supplement: S1 File — (DOC) [file pone.0330176.s001.doc]

**Figure 8. Performance comparison of different keyframe extraction models**

(a) Comparison of Training set

| Model | Precision | Recall | F1 Score | AUC |
| --- | --- | --- | --- | --- |
| Our | 0.820 | 0.763 | 0.793 | 0.799 |
| Reference [9] | 0.800 | 0.836 | 0.837 | 0.764 |
| Reference [12] | 0.766 | 0.782 | 0.803 | 0.795 |
| Reference [13] | 0.939 | 0.984 | 0.936 | 0.952 |

(b) Comparison of Test set

| Model | Precision | Recall | F1 Score | AUC |
| --- | --- | --- | --- | --- |
| Our | 0.834 | 0.858 | 0.836 | 0.818 |
| Reference [9] | 0.822 | 0.816 | 0.847 | 0.838 |
| Reference [12] | 0.819 | 0.808 | 0.789 | 0.795 |
| Reference [13] | 0.959 | 0.941 | 0.956 | 0.943 |

**Figure 9. Comparison of accuracy and redundancy of different keyframe extraction models**

(a) Accuracy rate

| / | 0 | 20 | 40 | 60 | 80 | 100 | 120 |
| --- | --- | --- | --- | --- | --- | --- | --- |
| Our | 0.715 | 0.858 | 0.907 | 0.931 | 0.967 | 0.984 | 0.996 |
| Reference [9] | 0.625 | 0.747 | 0.826 | 0.870 | 0.891 | 0.892 | 0.893 |
| Reference [12] | 0.302 | 0.648 | 0.748 | 0.818 | 0.850 | 0.852 | 0.854 |
| Reference [13] | 0.305 | 0.622 | 0.771 | 0.841 | 0.865 | 0.874 | 0.874 |

(b) Redundancy comparison

| / | 0 | 20 | 40 | 60 | 80 | 100 | 120 |
| --- | --- | --- | --- | --- | --- | --- | --- |
| Our | 0.695 | 0.155 | 0.070 | 0.058 | 0.047 | 0.035 | 0.020 |
| Reference [9] | 0.691 | 0.131 | 0.128 | 0.121 | 0.121 | 0.120 | 0.120 |
| Reference [12] | 0.687 | 0.484 | 0.428 | 0.335 | 0.344 | 0.316 | 0.314 |
| Reference [13] | 0.679 | 0.301 | 0.265 | 0.254 | 0.250 | 0.239 | 0.236 |

**Figure 10. Comparison of mAP and loss function for different models**

(a) Loss

| / | 0 | 20 | 40 | 60 | 80 | 100 | 120 |
| --- | --- | --- | --- | --- | --- | --- | --- |
| Our | 0.680 | 0.213 | 0.102 | 0.051 | 0.039 | 0.033 | 0.030 |
| Reference [14] | 0.688 | 0.351 | 0.213 | 0.138 | 0.110 | 0.102 | 0.102 |
| Reference [16] | 0.699 | 0.589 | 0.249 | 0.162 | 0.134 | 0.126 | 0.124 |
| Reference [17] | 0.687 | 0.411 | 0.272 | 0.201 | 0.182 | 0.162 | 0.162 |

(b) mAP

| / | 0 | 20 | 40 | 60 | 80 | 100 | 120 |
| --- | --- | --- | --- | --- | --- | --- | --- |
| Our | 0.309 | 0.850 | 0.939 | 0.972 | 0.995 | 0.995 | 0.998 |
| Reference [14] | 0.304 | 0.407 | 0.514 | 0.631 | 0.757 | 0.888 | 0.925 |
| Reference [16] | 0.314 | 0.486 | 0.752 | 0.776 | 0.794 | 0.804 | 0.808 |
| Reference [17] | 0.315 | 0.379 | 0.379 | 0.407 | 0.566 | 0.654 | 0.757 |

**Figure 11. Comparison of association accuracy and detection accuracy of different models**

(a) AssA comparison

| / | 0 | 20 | 40 | 60 | 80 | 100 |
| --- | --- | --- | --- | --- | --- | --- |
| Our | 0.296 | 0.389 | 0.503 | 0.720 | 0.844 | 0.995 |
| Reference [14] | 0.244 | 0.332 | 0.414 | 0.554 | 0.667 | 0.850 |
| Reference [16] | 0.130 | 0.222 | 0.357 | 0.554 | 0.667 | 0.715 |
| Reference [17] | 0.109 | 0.191 | 0.294 | 0.476 | 0.568 | 0.750 |

(b) DetA comparison

| / | 0 | 20 | 40 | 60 | 80 | 100 |
| --- | --- | --- | --- | --- | --- | --- |
| Our | 0.174 | 0.444 | 0.690 | 0.815 | 0.912 | 0.986 |
| Reference [14] | 0.054 | 0.074 | 0.395 | 0.460 | 0.513 | 0.698 |
| Reference [16] | 0.144 | 0.125 | 0.439 | 0.580 | 0.603 | 0.752 |
| Reference [17] | 0.008 | 0.017 | 0.310 | 0.652 | 0.784 | 0.819 |

**Figure 12. Comparison of application effects of different keyframe extraction models**

(a) Comparing of User Summary

| / | 0 | 5.0 | 15.0 | 25.0 | 35.0 | 45.0 | 55.0 |
| --- | --- | --- | --- | --- | --- | --- | --- |
| Our | 0.311 | 0.443 | 0.601 | 0.708 | 0.741 | 0.874 | 0.997 |
| Reference [9] | 0.307 | 0.325 | 0.365 | 0.508 | 0.591 | 0.649 | 0.803 |
| Reference [12] | 0.303 | 0.364 | 0.454 | 0.544 | 0.634 | 0.770 | 0.953 |
| Reference [13] | 0.304 | 0.350 | 0.451 | 0.487 | 0.591 | 0.681 | 0.764 |

(b) Comparing of missing rate

| / | 0 | 5.0 | 15.0 | 25.0 | 35.0 | 45.0 | 55.0 |
| --- | --- | --- | --- | --- | --- | --- | --- |
| Our | 0.000 | 0.039 | 0.071 | 0.095 | 0.152 | 0.213 | 0.230 |
| Reference [9] | 0.000 | 0.028 | 0.117 | 0.163 | 0.248 | 0.316 | 0.440 |
| Reference [12] | 0.000 | 0.035 | 0.092 | 0.180 | 0.284 | 0.362 | 0.440 |
| Reference [13] | 0.000 | 0.007 | 0.085 | 0.223 | 0.280 | 0.326 | 0.501 |

(c) Comparing of false positive rate

| / | 0 | 5.0 | 15.0 | 25.0 | 35.0 | 45.0 | 55.0 |
| --- | --- | --- | --- | --- | --- | --- | --- |
| Our | 0.000 | 0.028 | 0.046 | 0.063 | 0.074 | 0.120 | 0.223 |
| Reference [9] | 0.000 | 0.007 | 0.056 | 0.170 | 0.241 | 0.324 | 0.426 |
| Reference [12] | 0.000 | 0.007 | 0.028 | 0.081 | 0.170 | 0227 | 0.355 |
| Reference [13] | 0.000 | 0.008 | 0.177 | 0.259 | 0.334 | 0.415 | 0.454 |

**Figure 13. Comparison of extraction efficiency and robustness of different models**

(a) Accuracy rate

| / | 0 | 20 | 40 | 60 | 80 | 100 | 120 |
| --- | --- | --- | --- | --- | --- | --- | --- |
| Our | 0.992 | 0.950 | 0.903 | 0.874 | 0.817 | 0.793 | 0.773 |
| Reference [9] | 0.978 | 0.925 | 0.880 | 0.823 | 0.807 | 0.771 | 0.764 |
| Reference [12] | 0.972 | 0.921 | 0.876 | 0.799 | 0.736 | 0.718 | 0.720 |
| Reference [13] | 0.972 | 0.787 | 0.736 | 0.708 | 0.683 | 0.675 | 0.671 |

(b) Speed

| / | 0 | 20 | 40 | 60 | 80 | 100 | 120 |
| --- | --- | --- | --- | --- | --- | --- | --- |
| Our | 22.4 | 45.4 | 113.2 | 174.1 | 214.3 | 237.4 | 293.4 |
| Reference [9] | 12.0 | 24.7 | 65.1 | 65.9 | 78.1 | 125.3 | 141.3 |
| Reference [12] | 13.8 | 50.6 | 75.2 | 86.2 | 88.3 | 112.8 | 148.2 |
| Reference [13] | 6.9 | 22.9 | 54.6 | 56.9 | 59.5 | 101.2 | 129.3 |

Figure 14. Comparison of MAPE and IOU of different models

| / | 10 | 20 | 30 | 40 | 50 | 60 | 70 | 80 | 90 | 100 |
| --- | --- | --- | --- | --- | --- | --- | --- | --- | --- | --- |
| IOU key frame extraction | 0.45 | 0.12 | 0.26 | 0.33 | 0.34 | 0.41 | 0.53 | 0.71 | 0.72 | 0.83 |
| IOU Not extracted | 0.21 | 0.09 | 0.13 | 0.16 | 0.16 | 0.18 | 0.24 | 0.31 | 0.34 | 0.57 |
| MAPE key frame extraction | 0.95 | 0.85 | 0.76 | 0.72 | 0.62 | 0.60 | 0.33 | 0.24 | 0.18 | 0.11 |
| MAPE Not extracted | 0.97 | 0.84 | 0.79 | 0.69 | 0.66 | 0.66 | 0.66 | 0.45 | 0.41 | 0.36 |

**Figure 15. Comparison of missed detection rate and false detection rate of different object detection models**

(a) Comparison of missing rate

| / | Our | References[14] | References[16] | References[17] |
| --- | --- | --- | --- | --- |
| 1 | 0.23 | 0.31 | 0.26 | 0.32 |
| 2 | 0.23 | 0.35 | 0.32 | 0.34 |
| 3 | 0.27 | 0.30 | 0.40 | 0.38 |
| 4 | 0.21 | 0.33 | 0.32 | 0.29 |
| 5 | 0.27 | 0.37 | 0.28 | 0.34 |
| 6 | 0.26 | 0.38 | 0.37 | 0.34 |
| 7 | 0.28 | 0.40 | 0.31 | 0.34 |
| 8 | 0.28 | 0.27 | 0.38 | 0.35 |
| 9 | 0.28 | 0.38 | 0.27 | 0.26 |
| 10 | 0.22 | 0.36 | 0.29 | 0.30 |
| 11 | 0.26 | 0.29 | 0.30 | 0.26 |
| 12 | 0.21 | 0.26 | 0.37 | 0.26 |
| 13 | 0.26 | 0.38 | 0.30 | 0.37 |
| 14 | 0.25 | 0.40 | 0.30 | 0.36 |
| 15 | 0.22 | 0.32 | 0.27 | 0.26 |
| 16 | 0.26 | 0.29 | 0.37 | 0.33 |
| 17 | 0.26 | 0.27 | 0.28 | 0.29 |
| 18 | 0.28 | 0.29 | 0.39 | 0.39 |
| 19 | 0.24 | 0.40 | 0.41 | 0.39 |
| 20 | 0.25 | 0.30 | 0.41 | 0.41 |

(b) Comparison of false positive rate

| / | Our | References[14] | References[16] | References[17] |
| --- | --- | --- | --- | --- |
| 1 | 0.27 | 0.30 | 0.37 | 0.37 |
| 2 | 0.27 | 0.26 | 0.31 | 0.35 |
| 3 | 0.29 | 0.35 | 0.27 | 0.40 |
| 4 | 0.30 | 0.38 | 0.39 | 0.33 |
| 5 | 0.28 | 0.26 | 0.37 | 0.27 |
| 6 | 0.28 | 0.38 | 0.40 | 0.31 |
| 7 | 0.30 | 0.37 | 0.37 | 0.36 |
| 8 | 0.26 | 0.33 | 0.40 | 0.31 |
| 9 | 0.30 | 0.36 | 0.32 | 0.36 |
| 10 | 0.29 | 0.41 | 0.36 | 0.40 |
| 11 | 0.25 | 0.40 | 0.29 | 0.26 |
| 12 | 0.27 | 0.33 | 0.35 | 0.36 |
| 13 | 0.29 | 0.26 | 0.36 | 0.34 |
| 14 | 0.26 | 0.29 | 0.40 | 0.33 |
| 15 | 0.29 | 0.34 | 0.39 | 0.30 |
| 16 | 0.28 | 0.34 | 0.35 | 0.34 |
| 17 | 0.28 | 0.31 | 0.29 | 0.40 |
| 18 | 0.27 | 0.26 | 0.33 | 0.28 |
| 19 | 0.26 | 0.37 | 0.28 | 0.32 |
| 20 | 0.26 | 0.30 | 0.41 | 0.34 |
