# Supplementary material for: Protection and Analysis of Intangible Cultural Heritage Videos Based on Keyframe Extraction and Adaptive Weight Assignment
Source: PLoS One. 2025 Aug 29;20(8):e0330176. doi: 10.1371/journal.pone.0330176 (PMC12396677; doi:10.1371/journal.pone.0330176)
Supplement: Attachment — Submitted filename: Response to comments.doc [file pone.0330176.s003.doc]

Review Comments to the Author

Please use the space provided to explain your answers to the questions above. You may also include additional comments for the author, including concerns about dual publication, research ethics, or publication ethics. (Please upload your review as an attachment if it exceeds 20,000 characters)

Reviewer #1:

The manuscript presents methodologies documented in the literature with less significant extension or modification.

-For publication in a high-impact journal, the contribution must clearly advance the state of the art.

Reply: Thank you very much for your comments. The contribution of this study is shown below:

This study is contributed by the following three aspects: (1) It proposes an automatic analysis framework tailored for ICH video processing that effectively addresses practical challenges, such as high content redundancy, low processing efficiency, and difficulty identifying meaningful segments. (2) It improves the semantic understanding of video structures by optimizing the processes of keyframe extraction and object detection, thereby enhancing the accuracy and automation of content segmentation. (3) It offers a practical and scalable solution for archival management, structured indexing, and the visual presentation of ICH video resources. It demonstrates strong adaptability and broad potential for the preservation of digital heritage.

-The work must convincingly demonstrate what is new or unique compared to prior work.

The authors should explicitly articulate the novelty of their work.

Reply: Thank you very much for your comments. The novelty of this study has been explained as follows:

This study is innovated in the following three aspects: (1) It introduces a 3D ResNet-18-based keyframe extraction model that uses spatio-temporal convolution to detect semantic changes between video frames. Because it does not rely on static frame templates, the model enhances the understanding of unstructured video content. (2) Ratio-Net, an anchor-free object detection algorithm, is developed and integrated with an attention mechanism. This improves spatial region perception and enhances recognition of complex targets, eliminating the need for manually defined anchor boxes. Third, the study seamlessly integrates keyframe extraction and object detection into an optimized, unified framework for ICH videos. This addresses the current lack of domain-specific video analysis techniques in this field.

-The literature review section lacks a critical gap analysis that justifies the need for the present study. In addition, comparative analysis with more recent and advanced techniques is missing. Enhance the literature review to identify clear gaps and show how the proposed work addresses them, incorporating comparative study against state-of-the-art methods to presentt the improvements.

Reply: Thank you very much for your comments. The key gap analysis has been added to show the necessity of this study, and the solutions to these issues and the comparative analysis of the advanced techniques have been explained. Please see the revisions as follows:

In summary, although keyframe extraction and object detection methods have achieved success in certain scenarios, they still have limitations. First, approaches such as keyframe extraction and action template methods generally rely on scene priors, making them poorly suited for ICH videos, which tend to be loosely structured and semantically complex. Second, object detection models such as YOLOv8-QSD rely on anchor-based mechanisms that necessitate the manual configuration of various sizes and aspect ratios. This limits the flexibility of the model and its ability to generalize across different domains. Lastly, although 3D detection networks like VoPiFNet incorporate voxel-pixel fusion mechanisms, they are primarily intended for LiDAR data. Consequently, they exhibit modeling bias when applied to RGB videos and lack the ability to capture temporal features.

To overcome these limitations, this study introduces a 3D ResNet-18 structure based on shot boundaries to extract keyframes. This structure uses spatio-temporal convolutions to capture dynamic semantic transitions between video frames. This approach eliminates the need for static inter-frame features or predefined templates, thereby improving the analysis of unstructured video content. The study proposes Ratio-Net, an anchor-free object detection algorithm that incorporates an attention mechanism to improve spatial region perception, for object detection. Unlike anchor-based structures, such as YOLOv8, the proposed method eliminates anchor design constraints and enhances the accuracy and stability of recognizing irregular and low-texture targets in ICH videos. Additionally, this framework unifies optimization from video structure parsing to semantic recognition by integrating the keyframe extraction and object detection processes. This effectively addresses the current issues of fragmented workflows and weak robustness in cultural video processing.

-Consider integrating hybrid approaches, optimization strategies, or domain-specific enhancements that differentiate the work.

Reply: Thank you very much for your comments. The method proposed in the manuscript fully embodies integration and optimization for the video protection and analysis of intangible cultural heritage in the design. In terms of key frame extraction, a spatio-temporal convolution structure based on 3D ResNet-18 was adopted, which broke through the traditional static frame difference or template matching methods and improved the adaptability to intangible cultural heritage videos. In terms of object detection, the anchor-frameless algorithm Ratial-Net is proposed. Combined with FPN and the proportional regression strategy, it effectively improves the detection accuracy of low-texture objects and complex scenes. In addition, the method proposed in the manuscript has been specifically optimized for challenges such as complex movements, low-texture targets, and loose structures in intangible cultural heritage videos, enhancing its application potential in the protection and analysis of intangible cultural heritage videos. This method is not only applicable to the extraction and protection of key contents in intangible cultural heritage videos, but can also be extended to fields such as the digital archiving and analysis of cultural heritage.

-Authors should provide justification for choosing ResNet-18 over deeper or more lightweight alternatives (e.g., MobileNetV3, EfficientNet, Swin Transformers). Additionally, consider domain adaptation strategies such as fine-tuning on ICH-specific video datasets or introducing task-specific heads to improve performance in heritage data.

Reply: Thank you very much for your comments. The reasons for choosing ResNet-18 have been provided, and the domain adaptation strategies have been explained as follows:

**The reasons for choosing ResNet-18:**

To accurately obtain the temporal and spatial position information of the switching shots and the fade-in and fade-out shots, 3D ResNet-18 is selected as the backbone network for keyframe extraction in the study. ICH videos are usually loose in structure, slow in rhythm and sparse in semantics. The 3D ResNet-18 model effectively captures action changes and scene transitions without increasing excessive computational overhead through residual connections, which achieve robust extraction of spatio-temporal features. Compared to lightweight networks such as MobileNetV3 and EfficientNet, ResNet-18 strikes a better balance between feature expression ability and training stability. It is well-suited to the in-depth analysis requirements of cultural videos.

The manuscript has employed some domain adaptation strategies. Specifically, based on the semantic sparsity and loose structure characteristics of intangible cultural heritage videos, this study introduces domain adaptability strategies such as the 3D ResNet-18 structure based on shot boundary detection to model the spatio-temporal variation characteristics of long sequence videos. The anchor-free box detection algorithm Ratio-Net, which integrates ratio information and attention mechanism simultaneously, improves the recognition ability of weak texture and irregular targets and enhances the adaptability of the model in intangible cultural heritage scenarios.

-The paper reports very high accuracy metrics (e.g., 0.996 keyframe accuracy, 0.986 detection accuracy) but lacks an error analysis or qualitative examples of failure cases.

Reply: Thank you very much for your comments. The analysis of the failure cases has been added as follows:

However, in a few cases, the model may misjudge the boundaries if the semantic changes between consecutive shots are too subtle, or if occlusion interference occurs. This can result in redundancy or missed detection in keyframe extraction.

In Figure 11(b), there are significant differences among the DetA values of different models. The object detection algorithm with adaptive weight allocation achieves the highest DetA value, at 0.986. However, detection box drift or association failure may still occur in scenarios with extremely uneven lighting or severe target occlusion.

-Alternatively, demonstrating significant performance or efficiency improvements over existing methods could also be valuable.

Reply: Thank you very much for your comments. The comparative experiments have been conducted with representative existing methods, such as YOLOv8-qsd and VoPiFNet, on multiple ICH video samples to comprehensively evaluate the proposed method’s detection performance and computational efficiency. The results demonstrate significant improvements in key metrics such as AssA, DetA, and mAP, along with higher efficiency in terms of keyframe redundancy reduction and processing time. These findings highlight the superior performance and practical value of our method in the context of ICH video analysis.

-The contribution statement is not convincingly indepth and novel.

Reply: Thank you very much for your comments. The contribution of the manuscript has been clearly stated. As shown below:

This study is contributed by the following three aspects: (1) It proposes an automatic analysis framework tailored for ICH video processing that effectively addresses practical challenges, such as high content redundancy, low processing efficiency, and difficulty identifying meaningful segments. (2) It improves the semantic understanding of video structures by optimizing the processes of keyframe extraction and object detection, thereby enhancing the accuracy and automation of content segmentation. (3) It offers a practical and scalable solution for archival management, structured indexing, and the visual presentation of ICH video resources. It demonstrates strong adaptability and broad potential for the preservation of digital heritage.

This study is innovated in the following three aspects: (1) It introduces a 3D ResNet-18-based keyframe extraction model that uses spatio-temporal convolution to detect semantic changes between video frames. Because it does not rely on static frame templates, the model enhances the understanding of unstructured video content. (2) Ratio-Net, an anchor-free object detection algorithm, is developed and integrated with an attention mechanism. This improves spatial region perception and enhances recognition of complex targets, eliminating the need for manually defined anchor boxes. Third, the study seamlessly integrates keyframe extraction and object detection into an optimized, unified framework for ICH videos. This addresses the current lack of domain-specific video analysis techniques in this field.

-Clearly list the contributions and ensure each is supported by corresponding evidence in the results and discussion sections. Strengthen the discussion of results by connecting them directly to the claimed contributions.

Reply: Thank you very much for your comments. The contribution of the manuscript has been clearly stated. As shown below:

This study is contributed by the following three aspects: (1) It proposes an automatic analysis framework tailored for ICH video processing that effectively addresses practical challenges, such as high content redundancy, low processing efficiency, and difficulty identifying meaningful segments. (2) It improves the semantic understanding of video structures by optimizing the processes of keyframe extraction and object detection, thereby enhancing the accuracy and automation of content segmentation. (3) It offers a practical and scalable solution for archival management, structured indexing, and the visual presentation of ICH video resources. It demonstrates strong adaptability and broad potential for the preservation of digital heritage.

**The conclusion section has been rewritten, providing data that can support the above contributions. The revised conclusion is as follows:**

This study addressed common challenges in processing ICH videos, including high content redundancy, low processing efficiency, and difficulty rapidly extracting key segments. For the cultural video context, a new automated analysis framework was proposed. This framework enhanced semantic structure understanding by jointly optimizing keyframe extraction and object detection. In the keyframe extraction module, the 3D ResNet-18 model based on shot boundary detection performed well, achieving a precision of 0.939, a recall of 0.984, an F1 score of 0.936, and an AUC of 0.952. With a redundancy rate of only 0.02, it successfully preserved essential content and reduced data volume, meeting the practical needs of ICH video archiving and management. The anchor-free algorithm with adaptive weight allocation achieved optimal performance for object detection across multiple metrics. It produced a minimum loss of 0.03, a fast and stable mAP curve, and AssA and DetA values of 0.995 and 0.986, respectively. These results indicated strong adaptability to complex backgrounds and low-texture targets. Further experiments revealed that incorporating keyframe extraction significantly improved the IoU of object detection. Accuracy of the prediction box increased by 41.57%, reflecting substantial gains in efficiency and precision. In summary, the proposed framework was a feasible and effective technical solution for the structured analysis, management of content, and visual dissemination of ICH videos. It was highly adaptable and had great potential for practical application.

-Consider including future work section that outlines possible directions for extending the novelty.

Reply: Thank you very much for your comments. The future work section has been added, outlining the limitations and possible directions of this work.. As follows:

**6. Limitations and Future Work section**

Although the proposed method performs well in multiple experimental scenarios, it requires further validation to confirm its stability and adaptability in extreme lighting conditions, frequent shot transitions, and significant stylistic variations in regional ICH content. Additionally, the current model is designed for 2D video data only and does not address 3D heritage reconstruction or AR/VR-based interactive applications yet. Future research could focus on cross-domain generalization, multimodal integration (e.g., combining speech and motion cues), and adapting the framework for use in immersive environments. Studying these areas could make the framework more applicable in complex cultural contexts.

Reviewer #2:

Major issues

\# Section Comment & required action

M1 Methods §3.1 / Eq.(1–4) Derivations are unclear. Specify the exact feature vector produced by the CNN (dimensionality, layer) and justify the choice of λ and γ in Eq.(4). Provide an ablation showing robustness to these hyper-parameters.

Reply: Thank you very much for your comments.

**Eq.(1–4) Derivations have been modified, please see revisions as follows:**

In Equation (1), $\alpha$ represents the mixing coefficient, which controls the intensity with which the current frame is discriminated as the boundary of the shot. The larger the $\alpha$, the more inclined the model is to identify abrupt boundaries, making it suitable for scission-type shots. As $\alpha$ approaches smaller, it becomes more sensitive to gradual transition type shot switching. $I_{t}(x)$ denotes the pixel value of the image at position $x$ at time $t$ moment. $B_{t}$ and $F_{t}$ denote the pixel values of the first shot and the second shot, respectively.

**The exact feature vector generated by CNN and the choice of λ and γ in Eq.(4) has been explained as follows:**

The research uses a CNN to extract features from different video frames. More specifically, the output of the fourth residual block of the ResNet-18 network (i.e., the conv4 layer) is used for feature extraction. The output feature map (FM) of this layer has dimensions of 7×7×256. Calculating the L2 norm difference of FMs extracted from adjacent frames at this layer effectively measures the degree of semantic change between frames. This achieves preliminary detection of the no-shot boundary area.

In Equation (4), $\lambda$ is the magnification ratio for controlling the local difference degree. $\gamma$ is the relative threshold for controlling the difference from the global average. Together, they determine the shot boundary. The values are 1.2 and 0.25, which correspond to local mutation sensitivity and global difference tolerance, respectively. These values are obtained through the grid tone parameters of the training set and effectively balance the boundary recognition performance of shear and gradient shots.

**Furthermore, an ablation experiment related to parameters has been added as follows:**

Prior to the comparison experiments, hyperparameter ablation experiments are first performed to validate the hyperparameter $\lambda$ and the lens boundaries and to determine the effect of Equation (4) $\gamma$ on the keyframe extraction performance (accuracy and redundancy). The results in Table 1 show that when $\lambda$ and $\gamma$are in a reasonable range. Moreover, the overall performance of the model is stable. Among them, the best extraction performance is achieved when $\lambda$=1.2 and $\gamma$=0.5, with an accuracy of 0.991 and a redundancy of only 0.021. In contrast, when $\lambda$ is smaller (such as 1.0), the model is less sensitive to boundary changes. Furthermore, missing some boundaries results in decreased accuracy. When $\gamma$ is smaller (such as 0.15), the model is too sensitive to non-boundary areas, with more misjudgments and a significant increase in redundancy. Conversely, when $\gamma$ is too large, such as 0.30, the model tolerance is too high. This results in some weak but real shot transitions being missed, which slightly decreases accuracy.

Table 1 Results of hyperparameter ablation experiments

| $\lambda$ | $\gamma$ | Accuracy | Redundancy |
| --- | --- | --- | --- |
| 1.0 | 0.20 | 0.982 | 0.045 |
| 1.2 | 0.25 | 0.991 | 0.021 |
| 1.4 | 0.30 | 0.984 | 0.023 |
| 1.2 | 0.15 | 0.993 | 0.038 |

-M2 Methods §3.1 3-D ResNet-18 parameters are not given (input clip length, stride, pre-training, optimiser, learning-rate schedule, epochs, augmentation). Without these, the experiment cannot be reproduced.

Reply: Thank you very much for your comments. The parameters and other information related to 3D ResNet-18 have been added. As shown below:

For this study, the training parameters of the 3D ResNet-18 model are configured as follows: the length of the input video clip is set to 16 frames, and the stride is set to 1. Moreover, Pre-trained weights from the Kinetics-400 dataset are used for transfer learning. The Adam optimizer is employed with an initial learning rate of 1e-4, and a cosine annealing schedule is adopted for dynamic adjustment. The model is trained for 50 epochs. Data augmentation techniques, such as random cropping, horizontal flipping, and brightness perturbation, are applied during training to enhance generalization.

-M3 Methods §3.2 “Ratio-Net” is insufficiently described. Please: (a) release the network’s layer-wise architecture (filters, kernel sizes, activation), (b) clarify how ratio regression is normalised and how the three heads are weighted in the total loss.

Reply: Thank you very much for your comments. The detailed information of Ratio-Net has been added. As shown below:

Each branch of Ratio-Net has the following structure: the classification branch consists of three 3×3 CLs and one 1×1 output CL. All of these layers use the ReLU activation function. The bounding box regression branch has a similar structure, but its output channels are adjusted to 4×K. Among them, K is the number of categories, each of which outputs a set of box parameters (center coordinates, width, and height). The ratio information branch consists of two 3×3 CLs, followed by a Sigmoid normalization layer. This layer outputs the relative width and height ratios of the target. The Sigmoid function restricts the output to the range [0, 1] and multiplies it by the dimensions of the bounding box. This refines the boundaries and enhances the model's adaptability to targets of irregular shape.

**Information about RR has also been added. As shown below:**

The RR branch outputs the width-to-height ratio factors, which are then normalized using the Sigmoid function to constrain their values to the range [0, 1]. These ratios are multiplied by the predicted bounding box dimensions from the backbone network to determine the final box size. This improves the model's ability to adapt to irregularly shaped objects and prevents unstable predictions caused by unbounded regression values.

**The issue regarding the weighting of losses has been explained. As shown below:**

Furthermore, FPNS outputs at different layers consist of three branches: classification, regression, and ratio information. The loss weights for these branches are 0.4, 0.4, and 0.2, respectively. The classification and regression branches play a central role in the detection task, so they are assigned higher weights. The ratio information branch is primarily used to refine the size of the bounding box. To avoid its unstable impact on the overall optimization process, the weight is appropriately reduced in order to balance its auxiliary role with the stability of the overall training process.

-M4 Data The manuscript states that three public datasets are used, yet no train/val/test splits or licensing details are provided. Clarify whether original labels were modified. If custom splits are used, upload the split files.

Reply: Thank you very much for your comments. The processing of the data set has been further explained as follows:

To unify the experimental process and better align with the requirements of ICH-related video tasks, standardization is applied to some original labels in the ClipShots, TRECVID, and RAI datasets. During this process, all shot boundary annotations are converted into frame-level start and end indices using a consistent timestamp format based on frame counts. Ambiguous or inconsistent gradual transition labels are reclassified into clearly defined categories, such as "fade-in," "fade-out," or "dissolve." Additionally, samples with obvious labeling errors or missing annotations are corrected or excluded as appropriate. The experimental data are divided into the training set and the test set in an 8:2 ratio.

-M5 Experiments Figures 8-11 report only relative metrics. Report the absolute shot-boundary detection precision/recall/F1, mAP@0.5 and mAP@[0.5:0.95] for each dataset. Include standard deviation over at least three runs.

Reply: Thank you very much for your comments. More indicator results have been added as follows:

After verifying the superiority of the proposed method, it is tested on three datasets to confirm its robustness. After conducting multiple parallel experiments, the average value of the results was taken and the standard deviation was calculated, as shown in Table 2. The proposed method demonstrates high accuracy and stability across three public datasets. F1 scores reach 95.1%, 92.7%, and 93.7%, respectively, and standard deviations remain within ±0.5. This indicates that the model can generalize well under different video contents and structures. mAP@0.5 achieves the best performance on the RAI dataset at 90.4%. Under the more rigorous mAP@[0.5:0.95] metric, ClipShots and RAI reach 71.2% and 73.1%, respectively. This further demonstrates the adaptability and robustness of the method in multi-scale object detection and complex shot boundary recognition.

Table 2. Absolute performance metrics and standard deviations across three runs on each dataset

| Dataset | Precision (%) | Recall (%) | F1 Score (%) | mAP@0.5 (%) | mAP@[0.5:0.95] (%) |
| --- | --- | --- | --- | --- | --- |
| ClipShots | 93.8 ± 0.7 | 96.5 ± 0.5 | 95.1 ± 0.4 | 89.6 ± 0.6 | 71.2 ± 0.9 |
| TRECVID | 91.3 ± 0.8 | 94.2 ± 0.6 | 92.7 ± 0.5 | 88.1 ± 0.5 | 69.8 ± 0.7 |
| RAI | 92.5 ± 0.6 | 95.0 ± 0.4 | 93.7 ± 0.3 | 90.4 ± 0.4 | 73.1 ± 0.8 |

-M6 Baselines Some cited baselines (e.g., refs [9] & [12]) are for action recognition or surveillance summarisation, not key-frame extraction for general video. Justify their selection, or add more relevant baselines such as TransNet V2 (Sedláček et al., 2020) and SC-Cut (Baraldi et al., 2015).

Reply: Thank you very much for your comments. Some of the baseline methods cited in the manuscript (such as references [9] and [12]), although their original tasks were action recognition or video summarization, have strong capabilities in temporal modeling and semantic change detection, and are highly correlated with the modeling requirements for inter-frame dynamic changes in keyframe extraction. Therefore, they have certain reference value. Furthermore, "TransNet V2 (Sedla ček et al., 2020)" could not be found in the provided literature, and the second literature has been added. As shown below:

However, keypoint-based target detection is not very good at perceiving the internal information of the target object. Meanwhile, the accuracy of keypoint pairing is low due to the limitations of corner point information. Additionally, the detection algorithm that predicts the center point easily ignores global information and has poor adaptability to objects of special shapes [28-29].

[29] L. Baraldi, C. Grana, and R. Cucchiara, "A deep siamese network for scene detection in broadcast videos," Proc. 23rd ACM Int. Conf. Multimedia, pp. 1199–1202, Oct. 2015, doi: 10.1145/2733373.280631.

-M7 Statistical validity No statistical significance testing is shown. Apply paired t-test or Wilcoxon signed-rank on per-video metrics to demonstrate that improvements are not due to chance.

Reply: Thank you very much for your comments. The *p*-test results were supplemented in some experiments. As shown below:

The experimental results are verified by the *p*-test. The significance level *p*<0.05, indicating that the performance improvement of the proposed method is statistically significant and the results are reliable.

-M8 Writing quality There are many grammatical issues and over-claims (e.g., “accuracy was as high as 0.996”—this is dataset- and metric-specific). A thorough language edit is required.

Reply: Thank you very much for your comments. The language of the manuscript have been fully checked and revised.

-M9 Ethical statement The ethics/data-protection implications of processing indigenous cultural material are not addressed. Add a short discussion on consent, cultural sensitivity and data sharing.

Reply: Thank you very much for your comments. The ethical and cultural sensitivity statement has been added. As shown below:

ETHICAL AND CULTURAL SENSITIVITY STATEMENT

All of the video data related to ICH used in this study are obtained from publicly available datasets (e.g., ClipShots, TRECVID, and RAI). These datasets adhere to platform usage regulations and data-sharing permissions. Throughout the development and experimentation with the model, the research team has maintained a high level of respect for cultural sensitivity and intellectual property. All ICH content included in the study is publicly available and does not include unauthorized personal or community-specific footage. For future studies involving specific communities or cultural subjects, the research team will adhere strictly to ethical standards regarding informed consent, data protection, and cultural preservation. This ensures that all research activities are lawful, compliant, and respectful of cultural rights.

Minor issues

-Please replace “lens” with “shot” throughout to avoid confusion (e.g., Fig. 2 caption).

Reply: Thank you very much for your comments. The word "lens" has been replaced with "shot".

-Fig. 5: add resolution units to the tensor shapes.

Reply: Thank you very much for your comments. The resolution units have been added to the tensor shapes. As follows:

Note: Tensor shapes follow the format [C × H × W], with C = number of channels, H and W representing the spatial resolution in pixels.

-Eq.(9) appears twice (typo in numbering after Eq.(9)).

Reply: Thank you very much for your comments. The Eq.(9) has been revised to:

$\left(\begin{matrix} l=w\times l_{ratio} \\ r=w-l \\ t=h\times t_{ratio} \\ b=h-t \end{matrix}\right.$ (9)
