# Supplementary material for: Protection and Analysis of Intangible Cultural Heritage Videos Based on Keyframe Extraction and Adaptive Weight Assignment
Source: PLoS One. 2025 Aug 29;20(8):e0330176. doi: 10.1371/journal.pone.0330176 (PMC12396677; doi:10.1371/journal.pone.0330176)
Supplement: Peer review history

# Peer review: Decision Letter 0

**DOI:** 10.1371/journal.pone.0330176.r001

**Authors:** Ananth JP

20 May 2025

PONE-D-25-21647Protection and Analysis of Intangible Cultural Heritage Videos Based on Keyframe Extraction and Adaptive Weight AssignmentPLOS ONE

Dear Dr. Hou,

Thank you for submitting your manuscript to PLOS ONE. After careful consideration, we feel that it has merit but does not fully meet PLOS ONE’s publication criteria as it currently stands. Therefore, we invite you to submit a revised version of the manuscript that addresses the points raised during the review process.

Please submit your revised manuscript by Jul 04 2025 11:59PM. If you will need more time than this to complete your revisions, please reply to this message or contact the journal office at plosone@plos.org . When you're ready to submit your revision, log on to https://www.editorialmanager.com/pone/ and select the 'Submissions Needing Revision' folder to locate your manuscript file.

Please include the following items when submitting your revised manuscript:

- A rebuttal letter that responds to each point raised by the academic editor and reviewer(s). You should upload this letter as a separate file labeled 'Response to Reviewers'.
- A marked-up copy of your manuscript that highlights changes made to the original version. You should upload this as a separate file labeled 'Revised Manuscript with Track Changes'.
- An unmarked version of your revised paper without tracked changes. You should upload this as a separate file labeled 'Manuscript'.

If you would like to make changes to your financial disclosure, please include your updated statement in your cover letter. Guidelines for resubmitting your figure files are available below the reviewer comments at the end of this letter.

If applicable, we recommend that you deposit your laboratory protocols in protocols.io to enhance the reproducibility of your results. Protocols.io assigns your protocol its own identifier (DOI) so that it can be cited independently in the future. For instructions see: https://journals.plos.org/plosone/s/submission-guidelines#loc-laboratory-protocols . Additionally, PLOS ONE offers an option for publishing peer-reviewed Lab Protocol articles, which describe protocols hosted on protocols.io. Read more information on sharing protocols at https://plos.org/protocols?utm_medium=editorial-email&utm_source=authorletters&utm_campaign=protocols .

We look forward to receiving your revised manuscript.

Kind regards,

Ananth JP

Academic Editor

PLOS ONE

Journal requirements:

When submitting your revision, we need you to address these additional requirements.

1.  Please ensure that your manuscript meets PLOS ONE's style requirements, including those for file naming. The PLOS ONE style templates can be found at

https://journals.plos.org/plosone/s/file?id=wjVg/PLOSOne_formatting_sample_main_body.pdf   and

https://journals.plos.org/plosone/s/file?id=ba62/PLOSOne_formatting_sample_title_authors_affiliations.pdf

2. In your Methods section, please include additional information about your dataset and ensure that you have included a statement specifying whether the collection and analysis method complied with the terms and conditions for the source of the data.

3. Thank you for stating the following financial disclosure:

“The research is supported by 2024 Shaanxi Province Art and Science Planning Project: “Research on the Active Inheritance and Innovative Application of Folk Embroidery Techniques of the Qiang Ethnic Group in Southern Shaanxi in the Digital Age”, SYG2024011.”

Please state what role the funders took in the study.  If the funders had no role, please state: "The funders had no role in study design, data collection and analysis, decision to publish, or preparation of the manuscript."

If this statement is not correct you must amend it as needed.

Please include this amended Role of Funder statement in your cover letter; we will change the online submission form on your behalf.

4. We note that your Data Availability Statement is currently as follows: [All relevant data are within the manuscript and its Supporting Information files.]

Please confirm at this time whether or not your submission contains all raw data required to replicate the results of your study. Authors must share the “minimal data set” for their submission. PLOS defines the minimal data set to consist of the data required to replicate all study findings reported in the article, as well as related metadata and methods (https://journals.plos.org/plosone/s/data-availability#loc-minimal-data-set-definition).

For example, authors should submit the following data:

- The values behind the means, standard deviations and other measures reported;

- The values used to build graphs;

- The points extracted from images for analysis.

Authors do not need to submit their entire data set if only a portion of the data was used in the reported study.

If your submission does not contain these data, please either upload them as Supporting Information files or deposit them to a stable, public repository and provide us with the relevant URLs, DOIs, or accession numbers. For a list of recommended repositories, please see https://journals.plos.org/plosone/s/recommended-repositories.

If there are ethical or legal restrictions on sharing a de-identified data set, please explain them in detail (e.g., data contain potentially sensitive information, data are owned by a third-party organization, etc.) and who has imposed them (e.g., an ethics committee). Please also provide contact information for a data access committee, ethics committee, or other institutional body to which data requests may be sent. If data are owned by a third party, please indicate how others may request data access.

[Note: HTML markup is below. Please do not edit.]

Reviewers' comments:

Reviewer's Responses to Questions

**Comments to the Author**

1. Is the manuscript technically sound, and do the data support the conclusions?

The manuscript must describe a technically sound piece of scientific research with data that supports the conclusions. Experiments must have been conducted rigorously, with appropriate controls, replication, and sample sizes. The conclusions must be drawn appropriately based on the data presented. 

Reviewer #1: Partly

Reviewer #2: Yes

**********

2. Has the statistical analysis been performed appropriately and rigorously? 

Reviewer #1: N/A

Reviewer #2: Yes

**********

3. Have the authors made all data underlying the findings in their manuscript fully available?

The PLOS Data policy requires authors to make all data underlying the findings described in their manuscript fully available without restriction, with rare exception (please refer to the Data Availability Statement in the manuscript PDF file). The data should be provided as part of the manuscript or its supporting information, or deposited to a public repository. For example, in addition to summary statistics, the data points behind means, medians and variance measures should be available. If there are restrictions on publicly sharing data—e.g. participant privacy or use of data from a third party—those must be specified.

Reviewer #1: Yes

Reviewer #2: Yes

**********

4. Is the manuscript presented in an intelligible fashion and written in standard English?

PLOS ONE does not copyedit accepted manuscripts, so the language in submitted articles must be clear, correct, and unambiguous. Any typographical or grammatical errors should be corrected at revision, so please note any specific errors here.

Reviewer #1: Yes

Reviewer #2: Yes

**********

5. Review Comments to the Author

Please use the space provided to explain your answers to the questions above. You may also include additional comments for the author, including concerns about dual publication, research ethics, or publication ethics. (Please upload your review as an attachment if it exceeds 20,000 characters)

Reviewer #1: The manuscript presents methodologies documented in the literature with less significant extension or modification.

For publication in a high-impact journal, the contribution must clearly advance the state of the art.

The work must convincingly demonstrate what is new or unique compared to prior work.

The authors should explicitly articulate the novelty of their work.

The literature review section lacks a critical gap analysis that justifies the need for the present study.

In addition, comparative analysis with more recent and advanced techniques is missing.

Enhance the literature review to identify clear gaps and show how the proposed work addresses them, incorporating comparative study against state-of-the-art methods to presentt the improvements.

Consider integrating hybrid approaches, optimization strategies, or domain-specific enhancements that differentiate the work.

Authors should provide justification for choosing ResNet-18 over deeper or more lightweight alternatives (e.g., MobileNetV3, EfficientNet, Swin Transformers). Additionally, consider domain adaptation strategies such as fine-tuning on ICH-specific video datasets or introducing task-specific heads to improve performance in heritage data.

The paper reports very high accuracy metrics (e.g., 0.996 keyframe accuracy, 0.986 detection accuracy) but lacks an error analysis or qualitative examples of failure cases

Alternatively, demonstrating significant performance or efficiency improvements over existing methods could also be valuable.

The contribution statement is not convincingly indepth and novel.

Clearly list the contributions and ensure each is supported by corresponding evidence in the results and discussion sections.

Strengthen the discussion of results by connecting them directly to the claimed contributions.

Consider including future work section that outlines possible directions for extending the novelty.

Reviewer #2: Major issues

# Section Comment & required action

M1 Methods §3.1 / Eq.(1–4) Derivations are unclear. Specify the exact feature vector produced by the CNN (dimensionality, layer) and justify the choice of λ and γ in Eq.(4). Provide an ablation showing robustness to these hyper-parameters.

M2 Methods §3.1 3-D ResNet-18 parameters are not given (input clip length, stride, pre-training, optimiser, learning-rate schedule, epochs, augmentation). Without these, the experiment cannot be reproduced.

M3 Methods §3.2 “Ratio-Net” is insufficiently described. Please: (a) release the network’s layer-wise architecture (filters, kernel sizes, activation), (b) clarify how ratio regression is normalised and how the three heads are weighted in the total loss.

M4 Data The manuscript states that three public datasets are used, yet no train/val/test splits or licensing details are provided. Clarify whether original labels were modified. If custom splits are used, upload the split files.

M5 Experiments Figures 8-11 report only relative metrics. Report the absolute shot-boundary detection precision/recall/F1, mAP@0.5 and mAP@[0.5:0.95] for each dataset. Include standard deviation over at least three runs.

M6 Baselines Some cited baselines (e.g., refs [9] & [12]) are for action recognition or surveillance summarisation, not key-frame extraction for general video. Justify their selection, or add more relevant baselines such as TransNet V2 (Sedláček et al., 2020) and SC-Cut (Baraldi et al., 2015).

M7 Statistical validity No statistical significance testing is shown. Apply paired t-test or Wilcoxon signed-rank on per-video metrics to demonstrate that improvements are not due to chance.

M8 Writing quality There are many grammatical issues and over-claims (e.g., “accuracy was as high as 0.996”—this is dataset- and metric-specific). A thorough language edit is required.

M9 Ethical statement The ethics/data-protection implications of processing indigenous cultural material are not addressed. Add a short discussion on consent, cultural sensitivity and data sharing.

Minor issues

Please replace “lens” with “shot” throughout to avoid confusion (e.g., Fig. 2 caption).

Fig. 5: add resolution units to the tensor shapes.

Eq.(9) appears twice (typo in numbering after Eq.(9)).

**********

6. PLOS authors have the option to publish the peer review history of their article (what does this mean? ). If published, this will include your full peer review and any attached files.

If you choose “no”, your identity will remain anonymous but your review may still be made public.

**Do you want your identity to be public for this peer review?** For information about this choice, including consent withdrawal, please see our Privacy Policy .

Reviewer #1: No

Reviewer #2: **Yes: ** K L SHUNMUGANTHAN

**********

[NOTE: If reviewer comments were submitted as an attachment file, they will be attached to this email and accessible via the submission site. Please log into your account, locate the manuscript record, and check for the action link "View Attachments". If this link does not appear, there are no attachment files.]

While revising your submission, please upload your figure files to the Preflight Analysis and Conversion Engine (PACE) digital diagnostic tool, https://pacev2.apexcovantage.com/ . PACE helps ensure that figures meet PLOS requirements. To use PACE, you must first register as a user. Registration is free. Then, login and navigate to the UPLOAD tab, where you will find detailed instructions on how to use the tool. If you encounter any issues or have any questions when using PACE, please email PLOS at figures@plos.org . Please note that Supporting Information files do not need this step.

# Author comment: Author response to Decision Letter 1

**DOI:** 10.1371/journal.pone.0330176.r002

9 Jul 2025

The manuscript has been revised according to comments

Attachment Submitted filename: Response to comments.doc

# Peer review: Decision Letter 1

**DOI:** 10.1371/journal.pone.0330176.r003

**Authors:** Ananth JP

29 Jul 2025

Protection and Analysis of Intangible Cultural Heritage Videos Based on Keyframe Extraction and Adaptive Weight Assignment

PONE-D-25-21647R1

Dear Dr. Hou,

We’re pleased to inform you that your manuscript has been judged scientifically suitable for publication and will be formally accepted for publication once it meets all outstanding technical requirements.

Within one week, you’ll receive an e-mail detailing the required amendments. When these have been addressed, you’ll receive a formal acceptance letter and your manuscript will be scheduled for publication.

An invoice will be generated when your article is formally accepted. Please note, if your institution has a publishing partnership with PLOS and your article meets the relevant criteria, all or part of your publication costs will be covered. Please make sure your user information is up-to-date by logging into Editorial Manager at Editorial Manager®  and clicking the ‘Update My Information' link at the top of the page. For questions related to billing, please contact billing support .

If your institution or institutions have a press office, please notify them about your upcoming paper to help maximize its impact. If they’ll be preparing press materials, please inform our press team as soon as possible -- no later than 48 hours after receiving the formal acceptance. Your manuscript will remain under strict press embargo until 2 pm Eastern Time on the date of publication. For more information, please contact onepress@plos.org.

Kind regards,

Ananth JP

Academic Editor

PLOS ONE

Additional Editor Comments (optional):

Reviewers' comments:

Reviewer's Responses to Questions

**Comments to the Author**

1. If the authors have adequately addressed your comments raised in a previous round of review and you feel that this manuscript is now acceptable for publication, you may indicate that here to bypass the “Comments to the Author” section, enter your conflict of interest statement in the “Confidential to Editor” section, and submit your "Accept" recommendation.

Reviewer #1: All comments have been addressed

Reviewer #2: All comments have been addressed

**********

2. Is the manuscript technically sound, and do the data support the conclusions?

The manuscript must describe a technically sound piece of scientific research with data that supports the conclusions. Experiments must have been conducted rigorously, with appropriate controls, replication, and sample sizes. The conclusions must be drawn appropriately based on the data presented. 

Reviewer #1: Yes

Reviewer #2: Yes

**********

3. Has the statistical analysis been performed appropriately and rigorously? 

Reviewer #1: Yes

Reviewer #2: Yes

**********

4. Have the authors made all data underlying the findings in their manuscript fully available?

The PLOS Data policy requires authors to make all data underlying the findings described in their manuscript fully available without restriction, with rare exception (please refer to the Data Availability Statement in the manuscript PDF file). The data should be provided as part of the manuscript or its supporting information, or deposited to a public repository. For example, in addition to summary statistics, the data points behind means, medians and variance measures should be available. If there are restrictions on publicly sharing data—e.g. participant privacy or use of data from a third party—those must be specified.

Reviewer #1: Yes

Reviewer #2: Yes

**********

5. Is the manuscript presented in an intelligible fashion and written in standard English?

PLOS ONE does not copyedit accepted manuscripts, so the language in submitted articles must be clear, correct, and unambiguous. Any typographical or grammatical errors should be corrected at revision, so please note any specific errors here.

Reviewer #1: Yes

Reviewer #2: (No Response)

**********

6. Review Comments to the Author

Please use the space provided to explain your answers to the questions above. You may also include additional comments for the author, including concerns about dual publication, research ethics, or publication ethics. (Please upload your review as an attachment if it exceeds 20,000 characters)

Reviewer #1: (No Response)

Reviewer #2: The research is supported by 2024 Shaanxi Province Art and Science Planning

Project: “Research on the Active Inheritance and Innovative Application of Folk

Embroidery Techniques of the Qiang Ethnic Group in Southern Shaanxi in the Digital

Age”, SYG2024011.

**********

7. PLOS authors have the option to publish the peer review history of their article (what does this mean? ). If published, this will include your full peer review and any attached files.

If you choose “no”, your identity will remain anonymous but your review may still be made public.

**Do you want your identity to be public for this peer review?** For information about this choice, including consent withdrawal, please see our Privacy Policy .

Reviewer #1: No

Reviewer #2: **Yes: ** K L SHUNMUGANTHAN

**********

# Editor report: Acceptance letter

**DOI:** 10.1371/journal.pone.0330176.r004

**Authors:** Ananth JP

PONE-D-25-21647R1

PLOS ONE

Dear Dr. Hou,

I'm pleased to inform you that your manuscript has been deemed suitable for publication in PLOS ONE. Congratulations! Your manuscript is now being handed over to our production team.

At this stage, our production department will prepare your paper for publication. This includes ensuring the following:

* All references, tables, and figures are properly cited

* All relevant supporting information is included in the manuscript submission,

* There are no issues that prevent the paper from being properly typeset

You will receive further instructions from the production team, including instructions on how to review your proof when it is ready. Please keep in mind that we are working through a large volume of accepted articles, so please give us a few days to review your paper and let you know the next and final steps.

Lastly, if your institution or institutions have a press office, please let them know about your upcoming paper now to help maximize its impact. If they'll be preparing press materials, please inform our press team within the next 48 hours. Your manuscript will remain under strict press embargo until 2 pm Eastern Time on the date of publication. For more information, please contact onepress@plos.org.

You will receive an invoice from PLOS for your publication fee after your manuscript has reached the completed accept phase. If you receive an email requesting payment before acceptance or for any other service, this may be a phishing scheme. Learn how to identify phishing emails and protect your accounts at https://explore.plos.org/phishing.

If we can help with anything else, please email us at customercare@plos.org.

Thank you for submitting your work to PLOS ONE and supporting open access.

Kind regards,

PLOS ONE Editorial Office Staff

on behalf of

Dr. Ananth JP

Academic Editor

PLOS ONE
